# The LisH Domain-Containing N-Terminal Fragment is Important for the Localization, Dimerization, and Stability of Katnal2 in *Tetrahymena*

**DOI:** 10.3390/cells9020292

**Published:** 2020-01-25

**Authors:** Ewa Joachimiak, Ewa Waclawek, Michal Niziolek, Anna Osinka, Hanna Fabczak, Jacek Gaertig, Dorota Wloga

**Affiliations:** 1 Laboratory of Cytoskeleton and Cilia Biology, Nencki Institute of Experimental Biology PAS, 3 Pasteur, 02-093 Warsaw, Poland; e.joachimiak@nencki.edu.pl (E.J.); e.m.waclawek@gmail.com (E.W.); m.niziolek@nencki.edu.pl (M.N.); a.osinka@nencki.edu.pl (A.O.); h.fabczak@nencki.edu.pl (H.F.); 2 Department of Cellular Biology, University of Georgia, Athens, GA 30602, USA; jgaertig@uga.edu

**Keywords:** katanin, microtubules, glutamylation, protein dimerization, *Tetrahymena*, cilia

## Abstract

Katanin-like 2 protein (Katnal2) orthologs have a tripartite domain organization. Two highly conserved regions, an N-terminal LisH (Lis-homology) domain and a C-terminal AAA catalytic domain, are separated by a less conserved linker. The AAA domain of Katnal2 shares the highest amino acid sequence homology with the AAA domain of the canonical katanin p60. Katnal2 orthologs are present in a wide range of eukaryotes, from protists to humans. In the ciliate *Tetrahymena thermophila*, a Katnal2 ortholog, Kat2, co-localizes with the microtubular structures, including basal bodies and ciliary outer doublets, and this co-localization is sensitive to levels of microtubule glutamylation. The functional analysis of Kat2 domains suggests that an N-terminal fragment containing a LisH domain plays a role in the subcellular localization, dimerization, and stability of Kat2.

## 1. Introduction

Microtubules play crucial roles in numerous cellular processes, including intracellular transport, generation and maintenance of the cell shape and cell polarity, segregation of the chromosomes, and cell motility. To perform these functions, microtubules undergo rearrangement that includes cycles of assembly and disassembly, fragmentation, and the transport of short microtubule fragments. The microtubule severing enzymes, including katanin (p60), katanin-like proteins, spastin, and fidgetin, are important regulators of the organization of microtubules. These AAA domain (ATPases associated with diverse cellular activities)-containing enzymes locally destabilize the microtubule lattice to generate gaps and microtubule breakage, which leads to the shortening, fragmentation, or complete disassembly of the microtubule polymer. The short microtubule fragments can be transported and serve as seeds for new assembly (for a review, see [1]). Alternatively, gaps in the microtubule lattice can be repaired by the incorporation of GTP-bound tubulin, leading to lattice stabilization and an increase in the microtubule polymer mass [2]. 

Among the microtubule-severing enzymes, katanin p60, katanin-like protein 1, spastin, and fidgetin share a similar domain organization; an N-terminal microtubule-interacting and -trafficking (MIT) domain and a C-terminal catalytic AAA domain, which are separated by a non-conserved linker [1]. In contrast, the katanin-like 2 protein (also known as katanin p60 subunit A-like 2 or Katnal2), instead of a MIT domain, has an N-terminal LisH (Lis-homology) domain, followed by a short helical region that we will call a CTLH (C-terminal to LisH) by analogy to other LisH domain-containing proteins [3]. The role of the LisH domain in Katnal2 is unknown. Moreover, compared to other microtubule-severing proteins, our knowledge concerning Katnal2 activity and function is limited. 

Katnal2 proteins are evolutionarily conserved in most eukaryotic lineages [4]. In murine fibroblasts (NIH3T3), kidney epithelial cells (mIMCD3), and HeLa cells, Katnal2 localizes within the cytoplasm, where it partly overlaps with microtubules of the interphase network, mitotic spindle, midbody, and centrioles. Additionally, Katnal2 was detected in primary cilia along their entire length [5,6]. Similar Katnal2 localization was observed in *Xenopus* XL177 cells assembling primary cilia [7]. In the multiciliated cells of *Xenopus* embryonic epidermis, Katnal2 localizes to the basal bodies and along the axoneme of the motile cilia [7]. In the unicellular parasites *Trypanosoma brucei* and *Leishmania major*, ectopically expressed Katnal2 localizes to flagella, especially at the base and the tip, and its expression reduces the length of flagella [8]. The ShRNA-induced silencing of Katnal2 in mammalian cells results in the formation of additional centrioles, a multipolar mitotic spindle, defects in cytokinesis, and reduced ciliogenesis [5]. The assembly of fewer and shorter cilia was also observed in multiciliated cells of *Xenopus* embryonic epidermis with depleted Katnal2 [7]. In mice, Katnal2 is important at multiple stages of spermatogenesis [9].

Detailed analyses of *Xenopus* embryos with depleted Katnal2 have revealed abnormalities during embryonic development and organogenesis, including a reduced brain size [7]. Defects in brain development are in agreement with observations in mice showing that Katnal2 also plays a role in neurons, specifically in dendrite arborization [10]. Interestingly, in humans, Katnal2 mutations may be associated with autism [11,12,13,14].

The molecular mechanisms behind Katnal2 activity remain unknown. Until now, there have been no data showing that Katnal2 can sever microtubules in vitro [1]. The overexpression of human GFP-Katnal2 in HeLa cells did not change the microtubule signal, suggesting that Katnal2 does not sever microtubules [6]. On the other hand, in mammalian cells with depleted Katnal2, tubulin acetylation was elevated, suggesting the increased longevity of microtubules [5]. However, in *Tetrahymena* cells lacking Kat2—an ortholog of Katnal2—hyperacetylated microtubules were not observed and the phenotype of the knockout cells was not detectably altered [4]. Interestingly, when co-expressed in HEK293T cells, Katnal2 co-immunoprecipitates with δ-tubulin and ε-tubulin and co-localizes with these non-microtubular tubulins in murine spermatids [9].

To shed light on the molecular mechanism of action of Katnal2, we re-investigated the localization and properties of Kat2 in a ciliate *Tetrahymena thermophila*, focusing on the role of the LisH domain. The LisH domain is present in a number of proteins [15], and has been shown to mediate their homodimerization [16,17,18,19] and stability [3,20]. Mutations in LisH disturb the intracellular localization of some proteins, including Lis1, transducin β-like 1X (TBL1), and oral-facial-digital type 1 (OFD1) proteins, and reduce their half-life time [20]. 

Here, we find that in *Tetrahymena thermophila,* Kat2 predominates near the basal bodies and at the tips of cilia, and its LisH domain-containing N-terminal region plays a role in protein localization, stability, and dimerization. 

## 2. Materials and Methods

### 2.1. Tetrahymena Strains and Culture 

*Tetrahymena* cells were cultured in a standard SPP (super proteose peptone) medium [21] supplemented with an antibiotic-antimycotic mix at 1:100 (Sigma-Aldrich, St-Louis, MO, USA), with shaking at 30 °C. The wild-type CU428.2 strain was obtained from the *Tetrahymena* Stock Center (Cornell University, Ithaca, NY, USA). The paclitaxel-sensitive CU522 strain that carries a mutation (K350M) in the *BTU1* (β-tubulin 1) coding region was used for the introduction of transgenes, enabling protein overexpression (positive transformants were selected based on their resistance to paclitaxel [22]). The previously described *Tetrahymena* GFP-Ttll6A strain carries a transgene for the overproduction of a GFP-tagged truncated Ttll6A (tubulin tyrosine ligase like 6A) tubulin glutamylase elongase (GFP-Ttll6A M241-V292 [23,24]).

### 2.2. Cross-Linkers

Glutaraldehyde (25%, Polysciences Inc., Warrington, PA, USA) was diluted with water to a final concentration of 0.04% and added to an equal volume of a protein fraction. EDC (1-ethyl-3-(3-dimethylaminopropyl) carbodiimide hydrochloride (Thermo-Fisher Scientific, Rockford, IL, USA), a cell-impermeable, zero-length crosslinker was prepared just before use as a 200 mM solution in water. A cell-permeable EGS (ethylene glycol bis (succinimidyl succinate), Thermo-Fisher Scientific, Rockford, IL, USA) that forms a 12-atom cleavable spacer arm, was prepared as a 100 mM solution in DMSO, just before use.

### 2.3. Protein Tagging and Domain Analysis 

All PCR reactions were performed using Phusion HSII High Fidelity Polymerase (Thermo-Fisher Scientific Baltics, Vilnius, Lithuania), with CU428.2 genomic DNA as a template. The primers used are listed in Table S1. To overexpress Kat2-HA or Kat2-2V5 in the *BTU1* locus, the coding region of *KAT2* (TTHERM_00414230) was cloned using MluI and BamHI restriction sites into pMTT1-HA (MTT1, Metallothionein 1) and pMTT1-2V5 plasmids, both derived from pMTT1-GFP [23]. Mutations predicted to either abolish the ATPase activity of the AAA domain (E347Q) or prevent LisH domain-mediated dimerization (I33R, L37R) and silent mutations, enabling screening for the positive clones, were introduced into the *KAT2* coding region using overlapping PCR. For domain truncation analyses, fragments of the *KAT2* coding region were amplified with the addition of MluI and BamHI restriction sites, and cloned into the pMTT1-HA plasmid. A total of 15 μg of plasmid DNA was digested with ApaI and SacII to separate the targeting fragment from the plasmid backbone, precipitated onto DNAdel Gold Carrier Particles (Seashell Technology, La Jolla, CA, USA) according to the manufacturer’s instructions, and was biolistically transformed into CU522 cells. Transformants were selected for 3–4 days on SPP supplemented with 20 μM paclitaxel (BioShop, Burlington, ON, CanadaBio) at 30 °C.

To overexpress Kat2-HA in *Tetrahymena* cells also carrying a transgene for the overexpression of GFP-Ttll6A in the *BTU1* locus [23,24], the coding region of *KAT2* was cloned into a plasmid that enables the overexpression of C-terminally HA-tagged protein in the *MTT1* locus [25]. Approximately 15–20 μg of plasmid was used for the transformation. Transformants were selected for 3–4 days at 30 °C on SPP supplied with paromomycin at a final concentration of 70 μg/mL (Sigma-Aldrich, St-Louis, MO, USA).

To co-express full-length Kat2-2V5 and HA-tagged Kat2 truncations, the *KAT2* coding region was cloned into a plasmid that enabled the overexpression as C-terminally 2V5-tagged protein in the genomic location carrying adjacent *GRL3* (Granule lattice) and *GRL4* genes. In the macronuclear genome, the sequences encoding *GRL3* and *GRL4* coding regions are located on opposite DNA strands and are separated by about 1 kb. To overexpress Kat2-2V5 in the GRL3/GRL4 region, we amplified a 0.8 kb fragment of the *GRL4* gene, adding SacII and BglII restriction sites at 5′ and 3′ ends, respectively (primers in Table S1), and cloned it into a p4T2 vector carrying a neo2 cassette digested with SacII and BamHI. In parallel, the HA coding region and *BTU1* 3′UTR (untranslated region) were removed from the Kat2-HA-overexpressing plasmid using BamHI and XhoI sites, and were replaced by the 2V5 coding region, followed by 0.6 kb of 3′UTR of *BTU1* (transcription terminator) obtained from the native locus expression plasmid [26] using BamHI and EcoRV restriction enzymes and 1.1 kb of the *GRL3* gene amplified from the genomic DNA with the addition of EcoRV and XhoI sites at 5′ and 3′ ends, respectively (Table S1). Next, the GRL4-neo2 fragment was cloned into the pKat2-2V5-GRL3 vector using SacI and Cla I restriction sites. About 10–15 μg of the obtained transgene was introduced into cells overexpressing one of the katanin variants: a full-length Kat2-HA, I33R L37R mutant, or truncations, all from the *BTU1* locus under the *MTT1* promoter. Transformants were selected for 3–4 days at 30 °C on SPP supplied with paromomycin at a final concentration of 100 μg/mL (Sigma-Aldrich, St-Louis, MO, USA).

To express C-terminally 3HA-tagged Kat2 in the native locus, 2.2 kb of the coding region and a 1.7 kb fragment of the 3′UTR of *KAT2* were amplified and cloned into the appropriate plasmid, as previously described [27]. About 10 μg of the final plasmid was digested with MluI and XhoI and used for biolistic transformation. Transformants were selected for 3–4 days at 30 °C on SPP with 1.5 μg/mL CdCl_2_ and 100 μg/mL paromomycin and then grown under increasing paromomycin and decreasing CdCl_2_ concentrations to promote phenotypic assortment.

### 2.4. Immunofluorescence and Transmission Electron Microscopy

For immunofluorescence analyses, cells were handled as drops on coverslips and fixed as previously described [27,28]. The primary antibodies were used as follows: monoclonal mouse anti-HA.11 (cat. 901503, BioLegend, San Diego, CA, USA) 1:300; polyclonal rabbit anti-HA (C29F4, Cell Signaling Technology, Leiden, The Netherlands) 1:300; monoclonal rabbit anti-V5 (D3H8Q, Cell Signaling Technology, Leiden, The Netherlands) 1:1600; anti-centrin 20H5 (cat. 04-1624, Merck Millipore, Billerica, MA, USA) 1:300; concentrated monoclonal mouse anti-α-tubulin 12G10 (Developmental Studies Hybridoma Bank, Iowa University, Iowa City, IA, USA) 1:300; polyclonal rabbit anti-polyE antibody, developed and kindly provided by Gorovsky lab (University of Rochester, Rochester, NY, USA) [29] at a 1:2000 dilution; and monoclonal mouse anti-K antigen 10D12 antibody kindly provided by Dr. J. Frankel (University of Iowa, Iowa City, IA, USA) at a 1:50 dilution.

To analyze Kat2-HA localization at the ultrastructural level, the Kat2-HA-overexpressing cells were induced with 1 μg/mL CdCl_2_ for 3 h and processed for immunoanalysis with anti-HA monoclonal antibodies using either classical TEM or cryofixation methods, as previously described [27]. All samples were analyzed using a JEM 1200 EX transmission electron microscope (JEOL Co, Tokyo, Japan).

### 2.5. Western Blots

Protein fractions were isolated from Kat2-HA-overexpressing cells and wild-type cells as a negative control. The total protein extract was prepared as previously described [28]. The cytoskeletal proteins were isolated as previously described [23]. Unless indicated otherwise, for Western blots, proteins from 10^5^ cells (total fraction) or 20–40 μg of proteins (cytoskeletal proteins) were separated on 10% SDS-PAGE gels. The primary antibodies were used as follows: monoclonal mouse anti-HA at a 1:3000 dilution, concentrated mouse anti-α-tubulin 12G10 antibodies diluted to 1:40000, polyclonal rabbit anti-polyE antibody at a 1:20000 dilution, and anti-GFP (ab6556 Abcam, Cambridge, UK) at 1:60,000.

### 2.6. Microtubule Polymerization, Microtubule Binding Assay, Protein Cross-Linking and Immunoprecipitation

Tubulin was purified from wild-type *Tetrahymena* cells and polymerized as previously described [30]. To purify a full-length or truncated Kat2-HA, cells carrying an appropriate transgene were cultured in SPP and treated with 2.5 μg/mL CdCl_2_ for 3 h with shaking to induce overexpression. The HA-tagged proteins were purified from the cytosolic fraction using a resin with conjugated anti-HA antibodies (Pierce HA Epitope Tag Antibody Agarose conjugated, Thermo Scientific, Rockford, IL, USA), according to the manufacturer’s instructions. Purified proteins were eluted with 0.2 M glycine (pH 2.2), neutralized by the addition of 1 M Tris with a pH of 9.5 (to a final concentration of 10 mM), and ultrafiltered on Vivaspin 6 columns (Sartorius, Goettingen, Germany). The protein concentration was determined using a Pierce BCA Protein Assay Kit (Thermo Scientific, Rockford, IL, USA).

Microtubules were polymerized from purified tubulin in the PME buffer (80 mM PIPES, 1mM EGTA, 1 mM MgSO_4_, 20 µM paclitaxel, and 2× concentrated protease inhibitors), diluted to 1 mg/mL, and incubated with purified Kat2 at room temperature. After 30 min, samples were centrifuged on the glycerol cushion (60% glycerol in PME buffer) for 50 min at 100,000× *g* at 25 °C. Pellets of microtubules and supernatants were analyzed by Western blot analysis using anti-HA and anti-α-tubulin (12G10) antibodies.

To analyze the formation of Kat2 dimers, cells carrying appropriate transgenes were grown in SPP medium supplemented with 2.5 μg/mL CdCl_2_. After 4 h, cells were collected and cytoskeletal and soluble fractions were purified [23]. Equal amounts of proteins from overexpressing strains (100 μg) were incubated for 30 min with 0.02% glutaraldehyde on ice [31]. Ten (tubulin analysis) or 35 μg (Kat2 analysis) of proteins were separated on either 7% or 9% SDS-PAGE gels, transferred onto nitrocellulose, and probed with anti-HA or anti-α-tubulin (12G10) antibodies.

For EDC crosslinking, the cytoskeletal proteins were isolated from overproducing cells that were lysed on ice for 1 min with 0.5% Triton-X100 in Dryl’s solution (1 mM Na_2_HPO_4_, 1 mM NaH_2_PO_4_, 1.5 mM CaCl_2_, and 2 mM sodium citrate, pH 7.1) with protease inhibitors. After centrifugation for 10 min at 21,000× *g* at 4 °C, pellets (cytoskeletal proteins) and supernatants were collected, and 100 μg of proteins was resuspended in Dryl’s solution with protease inhibitors and incubated with 2.5 mM EDC for an hour at RT. The reaction was stopped by the addition of 1M Tris with a pH of 7.4. The presence of protein complexes was analyzed by Western blot analysis (as above).

For in vivo crosslinking, cells at the density 10^5^ cells/mL were grown for 4 h in SPP medium supplemented with 2.5 μg/mL CdCl_2_ and washed with warm Dryl’s solution, and the cells were incubated in Dryl’s solution supplemented with either 0.8 mM EGS or 0.8% DMSO (control) for 75 min. at 30 °C. After the isolation of the cytoskeletal and supernatant fractions, the presence of protein complexes was analyzed as described above.

To identify proteins that co-immunoprecipitate with Kat2-HA, wild-type (control) and *Tetrahymena* cells carrying the *MTT1-KAT2-HA* transgene were induced for 3 h (2.5 μg/mL CdCl_2_), washed with diluted (1:3) PBS, pelleted, and incubated on ice with 1% Triton-X-100 in PBS and protease inhibitors for 1 min. After centrifugation, the pelleted cytoskeletal proteins were washed with PBS with protease inhibitors, and the protein concentration was estimated. The cytoskeletal proteins were incubated with 5 mM EDC for 1 h at RT and the reaction was stopped by 1 M Tris-HCl buffer with a pH of 7.4. Next, cytoskeletal proteins were incubated with denaturation buffer (1% SDS in 10 mM Tris-HCl, pH 7.4), heated for 5 min at 95 °C, chilled on ice and diluted 10 times with non-denaturation buffer (50 mM Tris, pH 7.4, 1% Triton-X-100, 300 mM NaCl, and 5 mM EDTA) with protease inhibitors, and centrifuged (10 min at 16,000× *g* at 4 °C), and the supernatant (2–4 mg of proteins) was incubated overnight with Pierce HA Epitope Tag Antibody Agarose conjugated (Thermo Scientific, Rockford, IL, USA) at 4 °C, according to the manufacturer’s instructions. After washing, precipitated proteins were separated on the polyacrylamide gel and silver stained and were identified by mass spectrometry (Laboratory of Mass Spectrometry, Institute of Biochemistry and Biophysics, PAS, Warsaw, Poland).

### 2.7. Protein Sequence Analysis

The amino acid sequences of Katnal2 orthologs were identified in the NCBI database using *Tetrahymena thermophila* Kat2 and human Katnal2 sequences as baits. The sequences were aligned using the ClustalX2 program [32] and edited in the SeaView program [33]. Some predicted protein sequences were manually corrected (see Figure S2’s legend). The position of the LisH and AAA domains was predicted using SMART (www. http://smart.embl-heidelberg.de/, [34]). The 3D structure of LisH and an adjacent CTLH domain of *Tetrahymena* Kat2 (TTHERM_00414230) and human Katnal2 (XP_005258414) were predicted using the automated protein structure homology-modeling server (https://swissmodel.expasy.org/ [35,36]). 

## 3. Results

### 3.1. Kat2, an Ortholog of Mammalian Katnal2, Co-Localizes with Microtubular Structures 

Katnal2 orthologs are ~60 kDa evolutionarily conserved proteins with two characteristic features: (i) an N-terminal, LisH (Lis1-homology) domain, followed by a partly helical amino acid stretch, here called a CTLH (C-terminal to LisH domain), and (ii) a C-terminal AAA catalytic domain that is most similar to the AAA domain of the canonical microtubule-severing enzyme—katanin p60 (Figures S1 and S2). In all Katnal2 orthologs, the region between LisH-CTLH and AAA domains, the so-called linker, is poorly conserved (Figure S2).

Katnal2 orthologs are present in diverse ciliated species (OrthoDB, http://cegg.unige.ch/orthodb6). However, we were unable to identify a Katnal2 ortholog in *Caenorhabditis elegans*, a species with immotile sensory cilia. In the predicted proteomes of plants, such as *Arabidopsis thaliana*, *Oryza sativa*, and the moss *Physcomitrella patens*, besides the canonical katanin p60, there are AAA domain proteins that are most similar to the AAA domain of the human Katnal2. These plant AAA domain proteins have a limited homology to the Katnal2-type proteins within the CTLH region, but lack a LisH domain (Figure S2).

A *Tetrahymena thermophila* Katnal2 ortholog, Kat2 (TTHERM_00414230) [4], is composed of 539 amino acids. The 33-amino acid-long LisH domain is positioned between R25 and L57, and is followed by the 37 conserved amino acids of the CTLH domain (D58–K92). The conserved C-terminal region of Kat2 (V213–V539) encompasses the AAA catalytic domain (P279–S416) (Figure S1).

**Figure 1:**
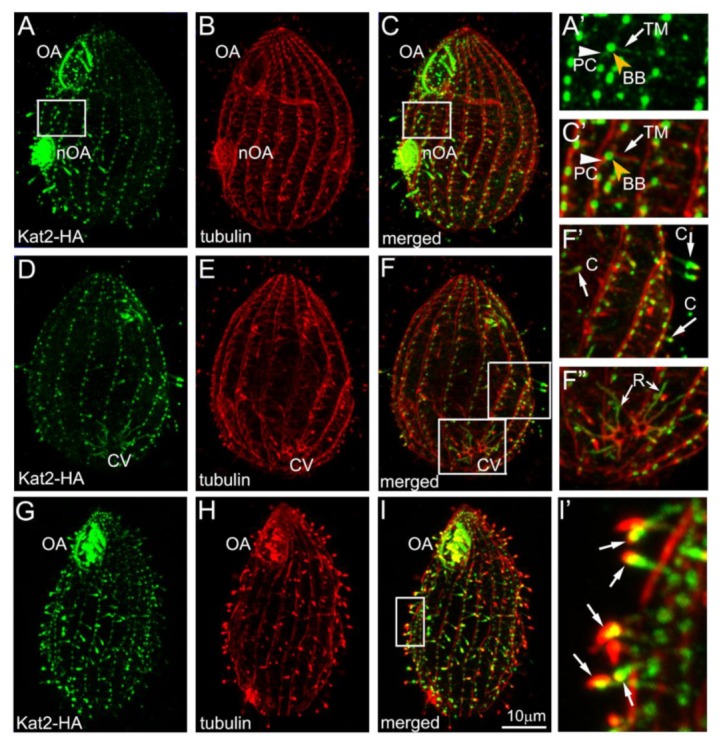
Kat2-HA localizes to microtubular organelles in *Tetrahymena*. Immunofluorescence confocal images of the ventral (**A**–**C**) and dorsal (**D**–**F**) sides of the Kat2-HA-overexpressing cells, showing a co-localization of Kat2-HA (**A**,**D**) with microtubular structures (**B**,**E**). (**C**,**F**) Merged images. Note a predominant localization of Kat2-HA in short growing cilia (**A**,**C**), especially at their distal ends (**F’**, arrows), near the basal bodies (**A’**,**C’**, yellow arrowhead), and weak staining along the transverse (**A’**,**C’**, white arrow) and postciliary microtubules (**A’**,**C’**, white arrowhead) and along microtubular radial rootlets of the contractile vacuole (**F”**, arrows). (**G**–**I**) Fluorescence confocal images showing Kat2-HA-overexpressing cells with short assembling cilia 30 min. after the experimental deciliation. Note the strong signal of Kat2-HA (**g**) in growing cilia, especially at their tips (**I’**, arrows). (**H**) tubulin staining, and (**I**) merged image. The white rectangles on (**C**), (**F)**, and (**I**) images show areas magnified in (**C’**), (**F’**), (**F”**), and (**I’**), respectively. Abbreviations: BB—basal body; C—cilium; CV—contractile vacuole; nOA—new oral apparatus; OA—oral apparatus; PC—postciliary microtubules; R—radial rootlets of the contractile vacuole; TM—transverse microtubules. Bar = 10 μm.

Kat2—expressed as a C-terminal 3HA-tagged fusion protein under the control of its own promoter—was detected in the cell body and at the tip of short, presumably assembling, somatic cilia (Figure S3B, arrow) and short assembling oral cilia in the newly developing oral apparatus (Figure S3A,B). Because the native level of Kat2 expression in *Tetrahymena* cells is very low (Figure S3C,D), we engineered a strain that expressed the Kat2-HA fusion protein under the control of a strong, cadmium-inducible promoter, *MTT1* [37]. Overexpressed Kat2-HA localized to cilia, especially shorter assembling cilia, where the protein was enriched at the distal tips (Figure 1A–C, D–F’, G–I’). Additionally, Kat2-HA was enriched near the basal bodies (Figure S3F–H’) and along microtubules of the radial rootlets of the contractile vacuole (Figure 1D–F”). A lower signal of Kat2-HA was detected along the transverse and postciliary microtubules that are located near each somatic basal body (Figure 1A’,C’). The *MTT1* promoter exhibited a low level of activity, even when Cd^2+^ was not added to the culture medium. At the basal level of the *MTT1* promoter expression (Figure S3C,D), Kat2-HA was apparent in cilia, again, especially in the assembling cilia and near basal bodies (Figure S3E–E”).

A similar localization pattern was observed in *Tetrahymena* cells overexpressing a E347Q Kat2-HA version (Figure S3I), carrying a substitution within the AAA domain, which in spastin abolishes the severing activity [38]. Therefore, the co-localization of Kat2 with microtubular structures does not require catalytic activity of the AAA domain. The overexpression of Kat2-HA E347Q did not change the cell phenotype.

**Figure 2:**
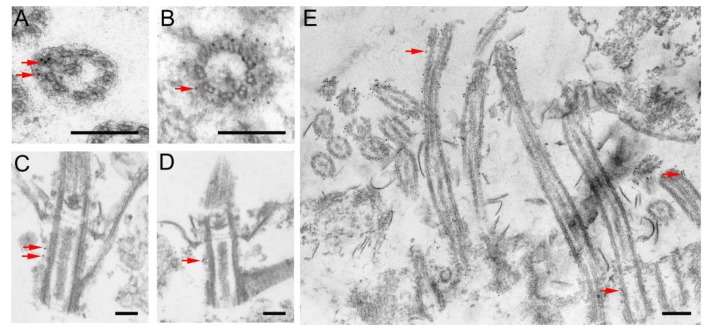
Immunogold TEM localization of Kat2-HA. Kat2-HA localizes near the outer microtubules in the axoneme (**A**,**E**) and basal bodies (**B**–**E**), of both somatic (**A**–**D**) and oral ciliary units (**E**). Cells were preserved either by cryofixation (**A**,**B**) or by chemical fixation (**C**–**E**). All (**A**,**C**,**D**) or exemplary gold grains (**B**,**E**) are indicated by red arrows. Overall, 57 and 80 gold grains were found decorating ciliary or basal body microtubules, respectively, in Kat2-HA-overexpressing cells, while only seven and five gold grains were seen near cilia or basal bodies, respectively, in the negative control wild-type cells. In total, 1300 sections of cilia and 317 sections of basal bodies were analyzed in control samples and 500 (80 decorated) and 100 (30 decorated) cilia and basal bodies sections in Kat2-HA-overexpressing cells, respectively. Bar = 200 nm.

An ultrastructural immunogold analysis of Kat2-HA-expressing cells induced with a low dose of cadmium chloride (1 μg/mL) revealed that Kat2-HA predominantly localized near the triplet microtubules of basal bodies (Figure 2B–D) and ciliary outer doublets (Figure 2A,E). Occasionally, gold grains were present near the cortical microtubules, including those supporting radial rootlets of the contractile vacuole (not shown).

### 3.2. Kat2-HA Preferentially Co-Localizes with Glutamylated Microtubules 

In *Tetrahymena* cells, the overexpression of a potent β-tubulin glutamylase elongase, Ttll6A, increases the levels of tubulin glutamylation, causing hyperstabilization of the cell body microtubules and the assembly of short cilia with structural defects [23,24]. Recently, we showed that Kat1—a *Tetrahymena* ortholog of a canonical katanin p60—is mislocalized in cells overexpressing GFP-Ttll6A, presumably because the level of glutamylation of microtubules is important for the localization of Kat1/p60 [27]. To determine whether the levels of tubulin glutamylation influence Kat2-HA localization patterns, we overexpressed Kat2-HA in cells that were either wild-type or co-expressed GFP-Ttll6A and had excessively glutamylated microtubules (Figures S4–S6) [23,24].

**Figure 3:**
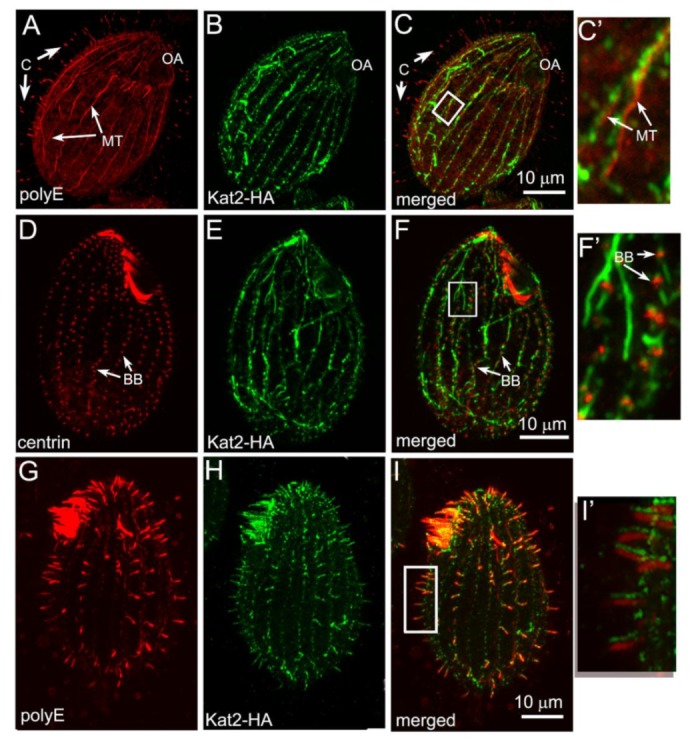
Increased tubulin glutamylation causes Kat2-HA mislocalization. Immunofluorescence confocal images of *Tetrahymena* cells induced with 2.5 μg mL^−1^ CdCl_2_ for 3 h to co-overexpress Kat2-HA and GFP-Ttll6A glutamylase, stained with anti-HA antibodies (**B**,**E**) and co-labeled with either anti-polyglutamylation polyE (**A**) or anti-centrin (**D**) antibodies. (**C**,**F**) Merged images. In cells overexpressing GFP-Ttll6A glutamylase, Kat2-HA was not detected in full-length cilia and near basal bodies, even in OA (please compare to Figure 1C), but co-localized with highly glutamylated subcortical microtubules that appeared as a result of the overexpression of GFP-Ttll6A (**A**,**C**). Note that cilia and basal bodies are present in cells co-overexpressing both enzymes (**A**,**C**,**D**,**F**). (**G**–**I’**) Kat2-HA is present in short, newly assembled cilia. Cells co-overexpressing Kat2-HA and GFP-Ttll6A glutamylase were deciliated and cultured in SPP medium supplemented with CdCl_2_ during cilia regeneration. Note that Kat2-HA (**H**) is present in short cilia containing highly glutamylated microtubules (**G**–**I**,**I’**). In (**I’**), the images obtained from two channels are slightly shifted to better visualize cilia staining. White rectangles on (**C**,**F**,**I**) show areas magnified in (**C’**,**F’**) and (**I’**), respectively. Abbreviations: BB—basal body; C—cilium; MT—bundles of microtubules; OA—oral apparatus. Bar = 10 μm.

Immunofluorescence showed that although cells with hyperglutamylated microtubules maintained cilia and basal bodies (Figure S6), Kat2-HA was rarely detectable in these structures (Figure 3A–F’). Instead, Kat2-HA was enriched along the hyperglutamylated cortical and cytoplasmic microtubules (Figure 3A, C–C’,Figure S4, Figure S6). In *Tetrahymena*, based on immunofluorescence, the levels of tubulin glutamylation were higher in the assembling than in the full-length cilia [4]. Indeed, when Kat2-HA and GFP-Ttll6A co-overexpressing cells were deciliated and allowed to regenerate cilia, Kat2-HA was enriched in the short assembling cilia (Figure 3G–I’). We concluded that in *Tetrahymena*, the pattern of localization of Kat2-HA is influenced by tubulin glutamylation, as it is in the case of Kat1/p60 [27].

### 3.3. LisH Domain Plays a Role in Kat2 Basal Body Targeting and Protein Stability 

GFP-Kat2 (N-terminal fusion), but not Kat2-GFP (C-terminal fusion), localizes in a diffused pattern in the cell body ([4 and our unpublished data]). Therefore, the GFP tag could affect the N-terminal region of Kat2, which includes LisH and CTLH; thus, this region may be important for the targeting of Kat2 to proper subsets of microtubular structures.

**Figure 4:**
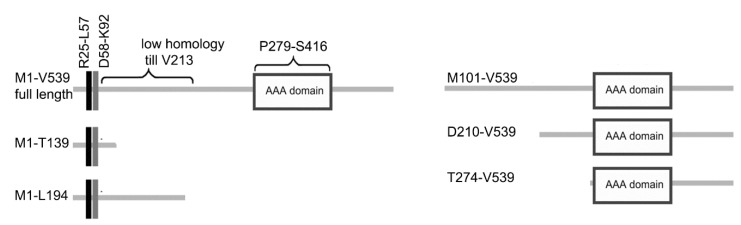
A graphical representation of the Kat2 domain organization and analyzed truncated variants. The black rectangle represents the LisH domain and the grey rectangle marks the position of the adjacent CTLH, whilst the white rectangle indicates the position of the AAA domain. Abbreviations: LisH—Lis-homology; CTLH—C-terminal to LisH.

To investigate the role of the LisH domain, and to identify other regions of Kat2 potentially involved in its subcellular localization, we analyzed the localization patterns of overexpressed HA-tagged truncated versions of Kat2 (Figure 4). 

Both a 139 amino acid-long N-terminal fragment containing LisH and CTLH (Kat2-HA M1-T139) and a slightly longer 194 amino acid fragment—also containing a region of limited homology (Kat2-HA M1-L194)—were targeted to the basal bodies, although the signal was much weaker compared to the one detected in cells expressing full-length Kat2-HA (Figure S7A,B,G). Additionally, a weak signal was observed in short, assembling oral cilia (Figure S7B). A Kat2 fragment that lacked LisH and CTLH (Kat2-HA M101-V539) was present in growing cilia (Figure S7C,G), but the signal near the basal bodies was weaker and more diffuse compared to cells expressing a full-length protein or LisH domain containing fragments (Figure S7H–J’). Kat2-HA M101-V539 was also present along the transverse and postciliary microtubules (Figure S7C). Moreover, in some cells, we observed Kat2 M101-V539-positive fiber-like structures within the cell body. A Kat2 truncation containing the AAA domain (Kat2-HA T274-V539) was present exclusively within the cell body, while Kat2-HA D210-V539 truncation containing the entire conserved C-terminal fragment co-localized with the basal bodies (Figure S7D–E,G). Therefore, the LisH and CTLH of Kat2 seem to play a role in targeting the basal bodies, although a contribution of the other Kat2 fragment(s) cannot be excluded.

To investigate if Kat2 fragments containing LisH and CTLH can bind to microtubules, we performed an in vitro microtubule-binding assay. The Kat2-HA M1-T139 and Kat2-HA M1-L194 truncations, the Kat2-HA E347Q mutant, and full-length Kat2-HA were purified from the cytosolic fraction of overexpressing cells using an anti-HA resin and were incubated with polymerized microtubules. Based on Western blots, full-length Kat2-HA was observed in the microtubule-bound fraction (Figure S8A). Similar results were obtained with Kat2-HA E347Q, carrying a mutation within the AAA domain. Under the same experimental conditions, both truncated fragments—Kat2-HA M1-T139 and Kat2-HA M1-L194—pelleted, even without microtubules, probably due to their oligomerization or aggregation. However, the amounts of truncated Kat2 in the pellets without microtubules were significantly lower than in the samples containing microtubules (Figure S8B). Therefore, we cannot exclude the possibility that LisH- and CTLH-containing fragments can bind to microtubules.

**Figure 5:**
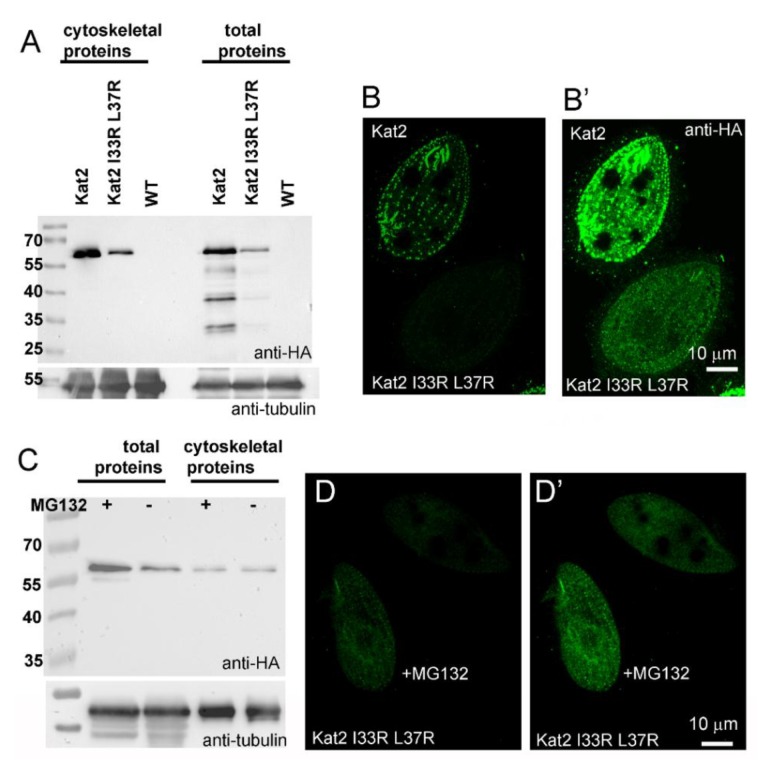
LisH domain plays a role in protein stabilization. (**A**) A Western blot comparative analysis of the levels of Kat2-HA and Kat2-HA I33R L37R in the cytoskeletal and total extracts of cells overexpressing one of these proteins. Corresponding fractions isolated from the wild-type cells (WT) were included as a control of the anti-HA antibody specificity. Tubulin (recognized by the 12G10 monoclonal antibody) was used as a loading control. (**B**,**B’**) Confocal images of the mixed cells overexpressing either Kat2-HA or Kat2-HA I33R L37R. Before fixation, cells expressing Kat2-HA were cultured for 10 min in SPP medium supplemented with an India ink and thus have dark food vacuoles. Note the lower signal of the mutated Kat2-HA I33R L37R protein compared to Kat2-HA (cell with dark vacuoles). (**B**,**B’**) show the same cells, but on (**B’**) cells, were overexposed to enhance the signal of Kat2-HA I33R L37R. (**C**) A Western blot analysis of the level of Kat2-HA I33R L37R in total and cytoskeletal extracts of cells overexpressing this protein and treated with MG132 (+), a 26S proteasome inhibitor, or with 0.4% DMSO (−). Tubulin was used as a loading control. Note that MG132 treatment increases the total amount of Kat2-HA I33R L37R, but does not visibly affect the amount of Kat2-HA I33R L37R in the cytoskeletal fraction. (**D**,**D’**) Immunofluorescence confocal images of cells overexpressing Kat2-HA I33R L37R for 6 h and treated with MG132 (+MG132) or with 0.4% DMSO (control, labeled with an India ink before fixation and thus having dark food vacuoles). Cells were mixed on the cover slip and fixed. (**D**,**D’**) show the same cells, but on (**D’**) cells, were overexposed to enhance the signal of Kat2-HA I33R L37R. Note an increased level of the Kat2-HA I33R L37R in MG132-treated cells. Bar = 10 μm.

In other proteins, LisH plays a role in protein dimerization (oligomerization) and stability [3,16,17,18,20]. The I33 and L37 residues of Kat2 correspond to the residues that, in other proteins, are important for LisH domain-mediated dimerization and stability [3,20]. To evaluate the significance of a LisH domain in Kat2 protein, we performed side-directed mutagenesis and substituted two amino acids in a LisH domain (I33, L37) by arginine residues (I33R, L37R). Overexpressed Kat2-HA I33R L37R was present at a lower level compared to overexpressed Kat2-HA, and was mostly detected within the cell body, but also near the basal bodies and in some cells in cilia (Figure 5A–B’ and Figure S7F). 

In the cases of LIS1, TBL1, OFD, and muskelin, mutations within LisH reduced the protein half-life time [3,20]. Therefore, we investigated whether inhibition of the 26S proteasome-dependent degradation would increase the levels of Kat2-HA I33R L37R. The proteasome inhibitor, MG132 (200 μM final), was added to the cell culture 2 h after induction of the protein overexpression. Side-by-side immunofluorescence of the MG132-treated cells and control cells treated with 0.4% DMSO showed that MG132 increased the levels of Kat2-HA I33R L37R-HA in *Tetrahymena* cells (Figure 5C–D’). Interestingly, MG132 treatment did not apparently increase the levels of Kat2-HA, Kat2-HA M1-T139, Kat2-HA M1-L194, or Kat2-HA M101-V539 (data not shown). Taken together, it is most likely that the LisH domain of Kat2 plays a role in protein stability and perhaps in co-localization with the basal bodies.

### 3.4. LisH Domain Mediates Kat2 Dimerization

**Figure 6:**
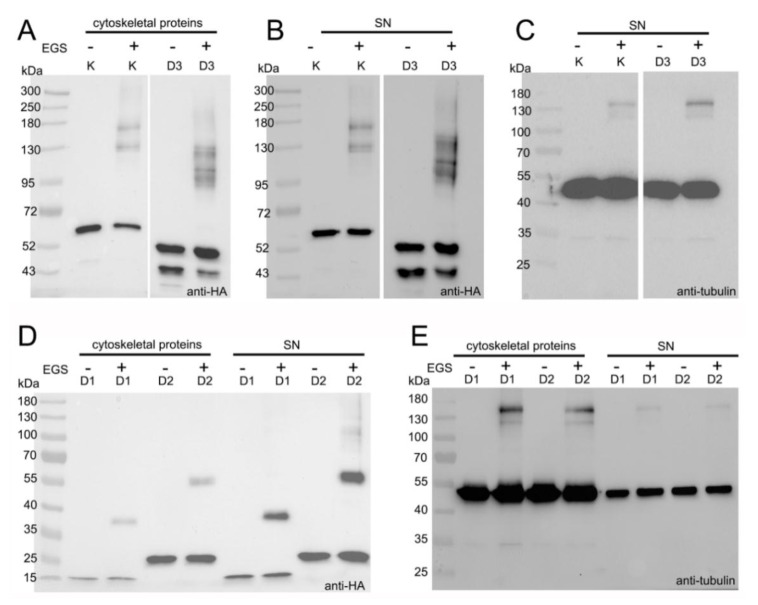
LisH domain mediates the formation of Kat2-HA complexes. (**A**–**E**) A Western blot-based identification of the HA-positive complexes (**A**,**B**,**D**) formed either in the cytoskeletal or supernatant (SN) fractions isolated from *Tetrahymena* cells overexpressing Kat2-HA (K), Kat2-HA M1-T139 (D1), Kat2-HA M1-L194 (D2), or Kat2-HA M101-V539 (D3) for 4 h. For in vivo crosslinking, overexpressing cells were resuspended in Dryl’s solution supplemented with 0.8 mM ethylene glycol bis(succinimidyl succinate) (EGS) (EGS+) and cultured for 75 min. at 30 °C. Control cells (EGS−) were incubated in Dryl’s medium supplemented with a corresponding concentration of DMSO (0.8%). (**B**,**C**) Whole blots are presented in Figure S9A–C.

To investigate whether LisH-CTLH mediates Kat2 dimerization, we tested whether Kat2 truncations can interact with each other and with full-length Kat2. *Tetrahymena* cells overexpressing (4 h) one of the following fusion proteins—Kat2-HA, Kat2-HA-I33R L37R, Kat2-HA M1-T139-HA, Kat2-HA M1-L194-HA, or Kat2-HA M101-V539-HA—were incubated for 75 min at 30 °C in Dryl’s solution supplemented with either 0.8% DMSO (control) or 0.8 mM EGS (ethylene glycol bis(succinimidyl succinate))—a cell-permeable crosslinker that forms a cleavable 12-atom spacer arm [39]. In vivo cross-linked Kat2 complexes in both cytoskeletal and supernatant fractions were analyzed by Western blot. In contrast to DMSO-treated controls, in fractions purified from Kat2-HA- or Kat2-HA I33R L37R-overexpressing cells treated with EGS, we detected additional HA-positive bands co-migrating with 130 and 180 kDa size markers (Figure 6A,B and Figure S9A,B). Bands of a similar size were detected using anti-tubulin antibodies (Figure 6C and Figure S9C). The predicted molecular mass of Kat2 is ~60 kDa. The molecular masses of α- and β-tubulin are both ~50 kDa, but in vivo, are likely to be higher due to tubulin posttranslational modifications. Because *Tetrahymena* Kat2-HA co-localizes with microtubules, the ~130 kDa HA-positive band could represent a Kat2-HA dimer or a tubulin monomer/Kat2-HA complex, while the ~180 kDa band could represent a complex composed of Kat2-HA and a tubulin heterodimer.

The hypothesis that Kat2-HA or Kat2-HA and tubulin are the main components of the detected complexes is supported by co-immunoprecipitation data. Mass spectrometry analyses revealed that, besides Kat2, only α- and β-tubulins were repeatedly detected when the full-length Kat2-HA was immunoprecipitated from the cytoskeletal fraction obtained from Kat2-HA-overexpressing cells (Table S2).

In the presence of EGS, several HA-positive bands of a higher molecular mass (~130–180 kDa) were also detected in the cytoskeletal and supernatant fractions isolated from cells overexpressing Kat2-HA M101-V539-HA, a 49 kDa LisH-less fragment of Kat2 (Figure 6A,B and Figure S9A,B). These bands were smaller when compared to the HA-positive bands detected in cells overexpressing a full-length protein. Interestingly, tubulin-positive complexes detected in Kat2-HA M101-V539-HA-overexpressing cells seemed to migrate in a similar way to tubulin-positive complexes in cells overexpressing full-length Kat2-HA (Figure 6C and Figure S9C). Together, these data suggest that at least some of the HA-positive bands likely correspond to Kat2-HA dimers/oligomers and that the lack of an N-terminal fragment does not exclude Kat2-HA dimerization/oligomerization—perhaps via an AAA domain. Interactions via an AAA domain could also explain why Kat2-HA I33R L37R forms complexes. Alternatively, HA-positive bands can correspond to the complexes composed of Kat2-HA and unknown protein(s) that were not identified in an immunoprecipitation assay.

In the presence of EGS, higher molecular mass HA-positive complexes of about 38 and 55 kDa, respectively (Figure 6D), were also detected in cells overexpressing either 16 kDa Kat2-HA M1-T139-HA (D1) or 22 kDa Kat2-HA M1-L194-HA (D2) truncations. Parallel analyses using anti-α-tubulin antibodies failed to detect tubulin-positive complexes with a molecular mass of 38 kDa (Figure 6E). Therefore, it is possible that (i) Kat2-HA M1-T139-HA and 22 kDa Kat2-HA M1-L194-HA formed dimers and that (ii) the N-terminal fragment containing LisH and CTLH can also mediate Kat2 interactions.

We performed similar analyses using crosslinkers that stabilize potential complexes in vitro: glutaraldehyde [31] and a zero-length crosslinker, EDC (1-ethyl-3-(3-dimethylaminopropyl) carbodiimide hydrochloride) [40]. In the first set of experiments, we induced *Tetrahymena* cells to overexpress one of the following polypeptides: Kat2-HA, Kat2-HA I33R L37R, Kat2-HA M1-T139, Kat2-HA M1-L194, or Kat2-HA M101-V539. Isolated cytoskeletal and supernatant proteins were incubated either for 30 min on ice with 0.02% glutaraldehyde, or for an hour with 2.5 mM EDC at room temperature. When cross-linked proteins were analyzed by Western blotting using anti-HA antibodies, we detected additional bands of about 130 and 180 kDa and larger, in samples isolated from cells expressing Kat2-HA or Kat2-HA I33R L37R, and about 38 and 55 kDa HA-positive bands in protein extracts obtained from cells expressing Kat2-HA M1-T139 and Kat2-HA M1-L194, respectively (Figure S9D–F and data not shown). Therefore, the use of in vitro and in vivo cross-linkers yields similar results. Surprisingly, higher-molecular mass HA-positive complexes were not detected if cytoskeletal and supernatant proteins isolated from cells overexpressing Kat2-HA M101-V539, a LisH-CTLH less fragment of Kat2, were incubated with glutaraldehyde or EDC (Figure S9D and data not shown). We hypothesize that once isolated, Kat2-HA M101-V539 complexes were unstable and prone to fast disintegration or formed aggregates that did not enter the gel.

**Figure 7:**
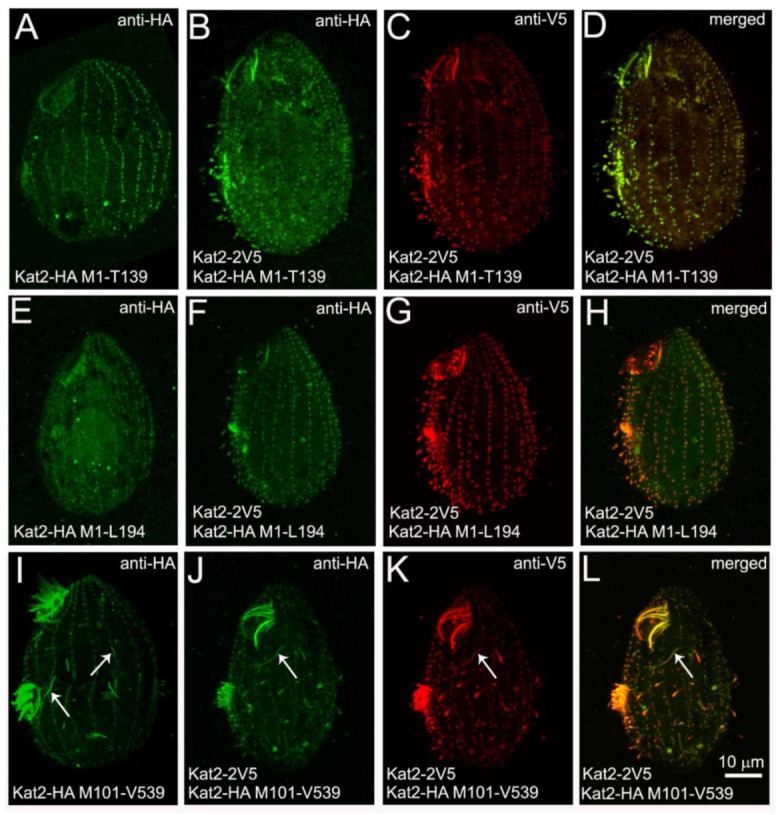
Co-overexpression of full-length Kat2-2V5 and HA-tagged Kat2 truncations causes the re-localization of proteins. Immunofluorescence confocal images of cells overexpressing either only C-terminally HA-tagged Kat2 truncations: (**A**) Kat2-HA M1-T139, (**E**) Kat2-HA M1-L194, or (**I**) Kat2-HA M101-V539, or co-overexpressing full-length Kat2-2V5 and HA-tagged Kat2 truncations: Kat2-HA M1-T139 (**B**–**D**), Kat2-HA M1-L194 (**F**–**H**), or Kat2-HA M101-V539 (**J**–**L**). Cells were stained with anti-HA (**A**,**B**,**E**,**F**,**I**,**J**) and anti-V5 (**C**,**G**,**K**) antibodies. (**D**,**H**,**L**) Merged images. Note that the pattern of co-overexpressed proteins overlaps (**D**,**H**,**L**). White arrows point to the fibers specifically present in cells overexpressing Kat2 M101-V539. Bar = 10 μm.

To further investigate if an N-terminal fragment containing LisH and CTLH can mediate the dimerization/interaction of Kat2, we tested whether LisH- and CTLH-containing truncations can interact with full-length Kat2 proteins. We co-overexpressed full-length Kat2-2V5 and truncated variants of Kat2 fused with HA tag. When expressed alone, Kat2-HA M1-T139 and Kat2-HA M1-L194 localized mainly near the basal bodies (Figure 7A,E and Figure S7A,B,H–I’). When HA-tagged Kat2-HA M1-T139 or Kat2-HA M1-L194 truncations were co-overexpressed with full-length Kat2-2V5, their localization overlapped. Both full-length specimens and truncations were present near basal bodies and in growing cilia (Figure 7B–D,F–H), suggesting an interaction between the HA-tagged Kat2 truncations and the full-length Kat2-2V5. Surprisingly, in co-overexpressing cells, the microtubules supporting radial rootlets of the contractive vacuoles were no longer decorated by full-length Kat2-2V5.

The co-overexpression of the full-length Kat2-2V5 and Kat2-HA M101-V539 fragment missing LisH and CTLH caused the partial mislocalization of full-length Kat2-2V5 and its association with fibers specifically decorated with Kat2-HA M101-V539 (Figure 7J–L, compared with Figure 7I, fiber-like structures indicated by white arrows).

Taken together, it seems that Kat2 can form complexes, and that these interactions can be mediated by both an N-terminal protein fragment containing LisH-CTLH domains and a C-terminal AAA domain.

## 4. Discussion

Among all katanin-related enzymes, the Katnal2 protein is the most enigmatic. Despite the high similarity within the AAA catalytic domain to the canonical katanin p60, the ability of the Katnal2 protein to sever microtubules was not demonstrated in vitro, while in vivo data are contradictory. While the overexpression of GFP-Katnal2 in HeLa cells did not visibly affect the microtubule network [6], the depletion of murine Katnal2 in NIH3T3 cells caused an increase in tubulin acetylation, suggesting an elevated microtubule stability [5]. In contrast, in *Tetrahymena*, the knockout of *KAT2* had no detectable effect on the phenotype of *Tetrahymena* cells, including the rate of cell proliferation, level of microtubule acetylation, and ciliary functions (our data not shown and Figures S3–S5 in [4]). Moreover, we did not observe a reduction of the microtubule signal upon the overexpression of Kat2-HA. However, it is possible that the impact of Kat2 on microtubules is subtle or highly spatially restricted. Therefore, it remains to be established whether *Tetrahymena* Kat2 and orthologs function as microtubule-severing proteins or/and play the role of microtubule-interacting proteins, affecting microtubule dynamics in a severing-independent manner.

Unlike other microtubule-severing proteins, Katnal2 orthologs have a LisH domain instead of a MIT domain, followed by a short conserved helical region in their N-terminal ends. LisH is a conserved alpha-helical domain present in numerous eukaryotic proteins [15,20] and was shown to mediate protein subcellular localization, dimerization (oligomerization), and stability. For example, in LIS1, an N-terminal region containing LisH, along with the region predicted to form a coiled-coil region, are required for protein homodimerization [16,41]. Mutations within the LisH domain of LIS1, TBL1, and OFD1 cause protein mislocalization and reduce their stability [20]. Similarly, the LisH domain of FOP1 (fibroblast growth factor receptor 1 (FGFR1) oncogene partner) and muskelin is involved in protein dimerization [3,17], and the LisH-containing fragment is also important for FOP1 localization to the centrosome [17].

In *Tetrahymena*, Kat2 expressed as N-terminal GFP fusion did not co-localize with the microtubular structures [4], while Kat2-GFP (our unpublished data) and Kat2-HA proteins co-localized with microtubular structures, including cilia and basal bodies. Therefore, it is likely that GFP positioned at the N-terminal end interferes with Kat2 targeting—either by changing protein conformations or by affecting interactions with other proteins. We have shown that fragments of Kat2 containing LisH and CTLH are targeted to the basal bodies, while Kat2 lacking the N-terminal region containing LisH and CTLH is partly mislocalized. Therefore, LisH domain-containing fragments could play a role in the targeting of Kat2 to the basal bodies. Basal bodies are homologous to the centrioles of the centrosome. The centrosomal localization of FOP requires an 80-amino acid region containing LisH [17]. On the other hand, the centrosomal localization of another LisH-containing protein, OFD1, is not influenced by LisH [42].

When co-expressed with full-length Kat2, localization of the N-terminal LisH and CTLH domain-containing fragments resembled the localization of a full-length protein, suggesting an interaction between Kat2 and the truncated version, most likely mediated by the LisH-dependent dimerization. Therefore, in Kat2—like in other proteins—LisH and CTLH may mediate protein dimerization. On the other hand, in the presence of the truncation that lacks an N-terminal fragment, the co-expressed, full-length Kat2 is partly mislocalized and resembles the localization of the co-expressed truncated form. Based on the obtained data, we hypothesize that the N-terminal end is not the sole region that enables the formation of Kat2 complexes, but that the C-terminal AAA domain also mediates protein interactions, as is the case of other AAA domain-containing proteins [43,44], and as has been suggested for Katnal2 [5]. If so, Katnal2 proteins may form complexes via both the LisH–CTLH domains and via the AAA domain. It remains to be determined whether these interactions occur simultaneously or are mutually exclusive. 

Finally, our observations suggest that Kat2 with mutations in LisH is prone to degradation. Structural studies showed that I15R and L19R substitutions within the LisH domain of LIS1 (see Figure S2 [20])—or the corresponding amino acid residues, C180 and F184, in muskelin [3]—affect protein dimerization and reduce their half-life. Similarly, when the corresponding amino acid residues, I33 and L37 were mutated in Kat2, the mutant protein was detected at a very low level and the amount of mutated protein increased when the cells were treated with an inhibitor of the 26S proteasome. Therefore, an LisH-dependent formation of Kat2 dimers or oligomers would possibly reduce the rate of protein degradation. 

In *Xenopus* embryos with depleted Katnal2, the multiciliated epidermis cells assembled fewer and shorter motile cilia [7]. In contrast, in *Trypanosoma brucei* and *Leishmania major*, mildly shortened flagella were observed in the cells that ectopically expressed Katnal2, while the RNAi-based reduction of the Katnal2 level had no obvious effect on the length of flagella in *Trypanosoma* [8]. In murine cells assembling primary cilia, both the depletion and overexpression of Katnal2 reduced ciliogenesis [5]. *Tetrahymena* cells, either overexpressing or lacking Kat2, assembled cilia in a similar number and of a similar length to wild-type cells (our unpublished data).

Interestingly, Kat2 expressed under the control of the native promoter migrates more slowly in SDS-PAGE gel compared to overexpressed Kat2. It is tempting to speculate that, under native conditions, Kat2 is post-translationally modified and—in the case of overexpressed Kat2—the amount of protein is too large to be effectively modified. If so, the unmodified Kat2 might be inactive or not fully active, and thus has no apparent effect on the cell phenotype.

Like in *Trypanosoma* and *Leishmania*, *Tetrahymena* Kat2 is most prominent at the cilia tip. It is possible that Kat2 may regulate the cilium length by modulating the dynamics of the plus ends of ciliary microtubules. Here, we have shown that Kat2 preferentially co-localizes with glutamylated microtubules. In cilia and flagella, only B-tubules of the peripheral doubles are highly glutamylated [45,46,47]. Therefore, Kat2 may regulate the elongation/dynamics of the plus end of B-tubules.

## Supplementary Materials

The following are available online at https://www.mdpi.com/2073-4409/9/2/292/s1. Figure S1. The analyses of the LisH and CTLH domains in human Katnal2 and *T. thermophila* ortholog, Kat2. Figure S2. A multiple alignment of Katnal2 ortholog sequences. Figure S3. A localization of Kat2-3HA expressed under the control of the native gene promoter. Figure S4. Partial co-localization of the overexpressed GFP-Ttll6A glutamylase and Kat2-HA. Figure S5. Comparative Western blot analysis of the level of tubulin glutamylation in cells co-overexpressing Kat2-HA and tubulin glutamylase, GFP-Ttll6A. Figure S6. The elevated level of tubulin glutamylation in cells co-overexpressing Kat2-HA and GFP-Ttll6A alters Kat2-HA localization. Figure S7. Immunolocalization of Kat2-HA truncations. Figure S8. An in vitro microtubule binding assay. Figure S9. The LisH domain plays a role in the formation of Kat2 complexes. Table S1. Primers used to amplify fragments of the genomic DNA. Table S2. Proteins that co-immunoprecipitate with Kat2-HA.
