# Supplementary material for: The LisH Domain-Containing N-Terminal Fragment is Important for the Localization, Dimerization, and Stability of Katnal2 in Tetrahymena
Source: Cells. 2020 Jan 25;9(2):292. doi: 10.3390/cells9020292 (PMC7072489; doi:10.3390/cells9020292)
Supplement: Supplementary file 1 [file cells-09-00292-s001.pdf]

Supplementary data

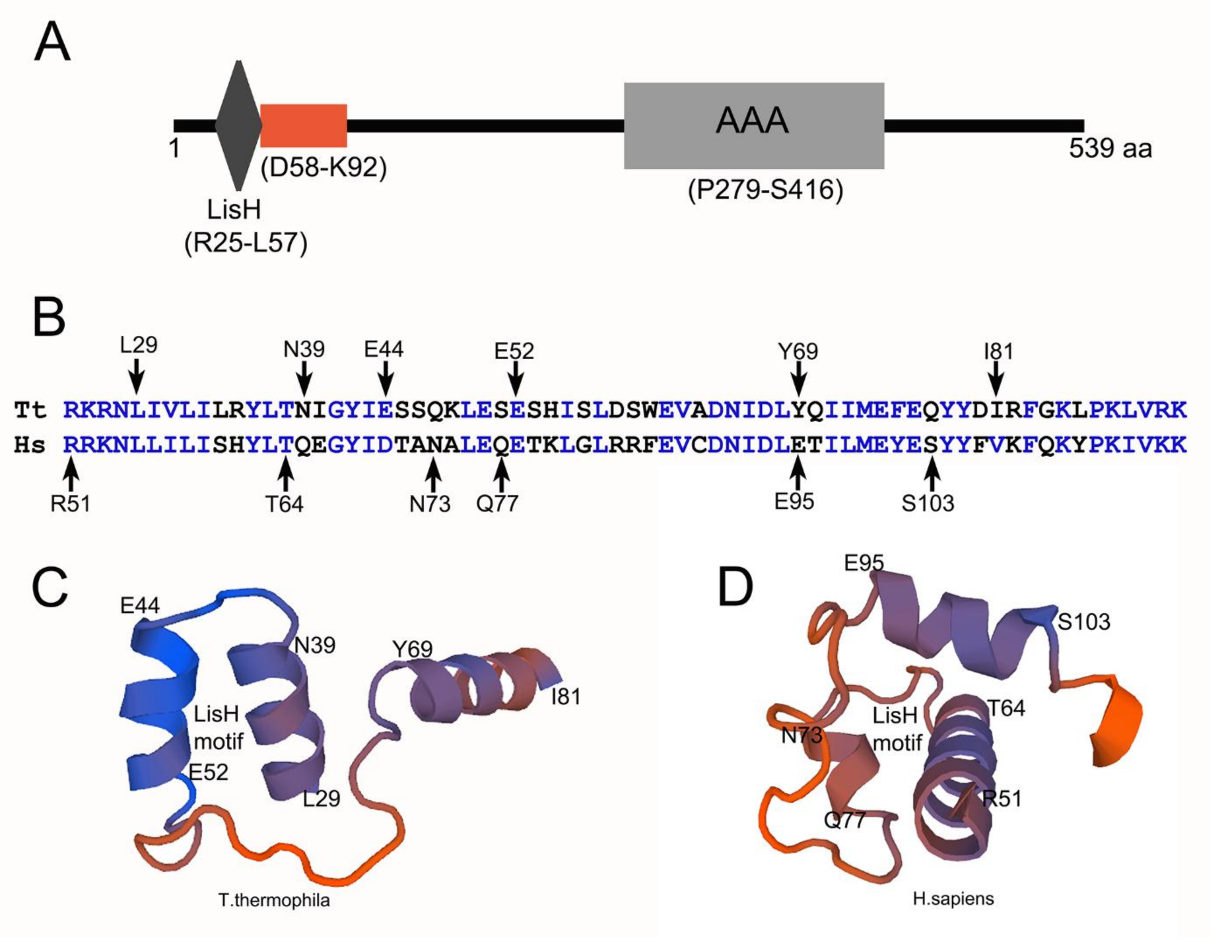

Figure S1. The analyses of the LisH and CTLH domains in human Katnal2 and *T. thermophila* ortholog, Kat2. (**a**) Schematic representation of domains in *T. thermophila* Kat2: LisH domain (black rhombus), CTLH (orange rectangle) and AAA domain (grey rectangle). The position of first and last amino acids of the domains are indicated. (**b**, **c**) An alignment and a 3D model of an N-terminal fragment of the *Tetrahymena* Kat2 (XP_001007193, TTHERM_00414230) and human Katnal2 (XP_005258414.1) containing a LisH domain and an adjacent CTLH as predicted, using an automated protein structure homology-modelling server (https://swissmodel.expasy.org/). Numbers indicate the initial and final (margin) amino acids of the predicted helices. Identical or similar amino acid residues in the corresponding aligned fragments of the *Tetrahymena* and human proteins are shown in blue.

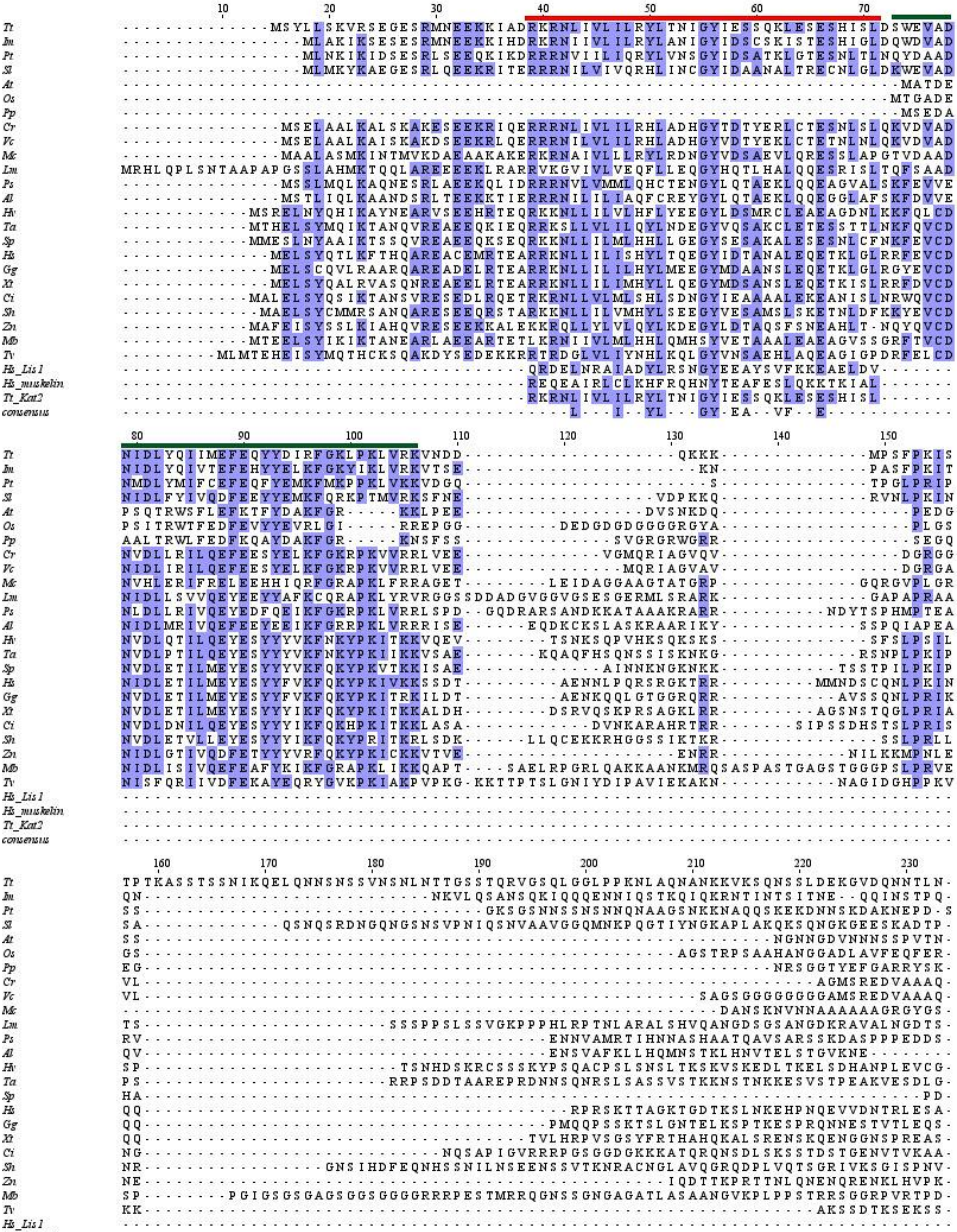
Tt
Bm
Pt
Sl
At
Os
Pp
Cr
Vc
Mc
Lm
Ps
Al
Hv
Ta
Sp
Hs
Gg
Xt
Ci
Sh
Zn
Mb
Tv
Hs_Lis1
Hs_muskelin
Tt_Kat2
consensus

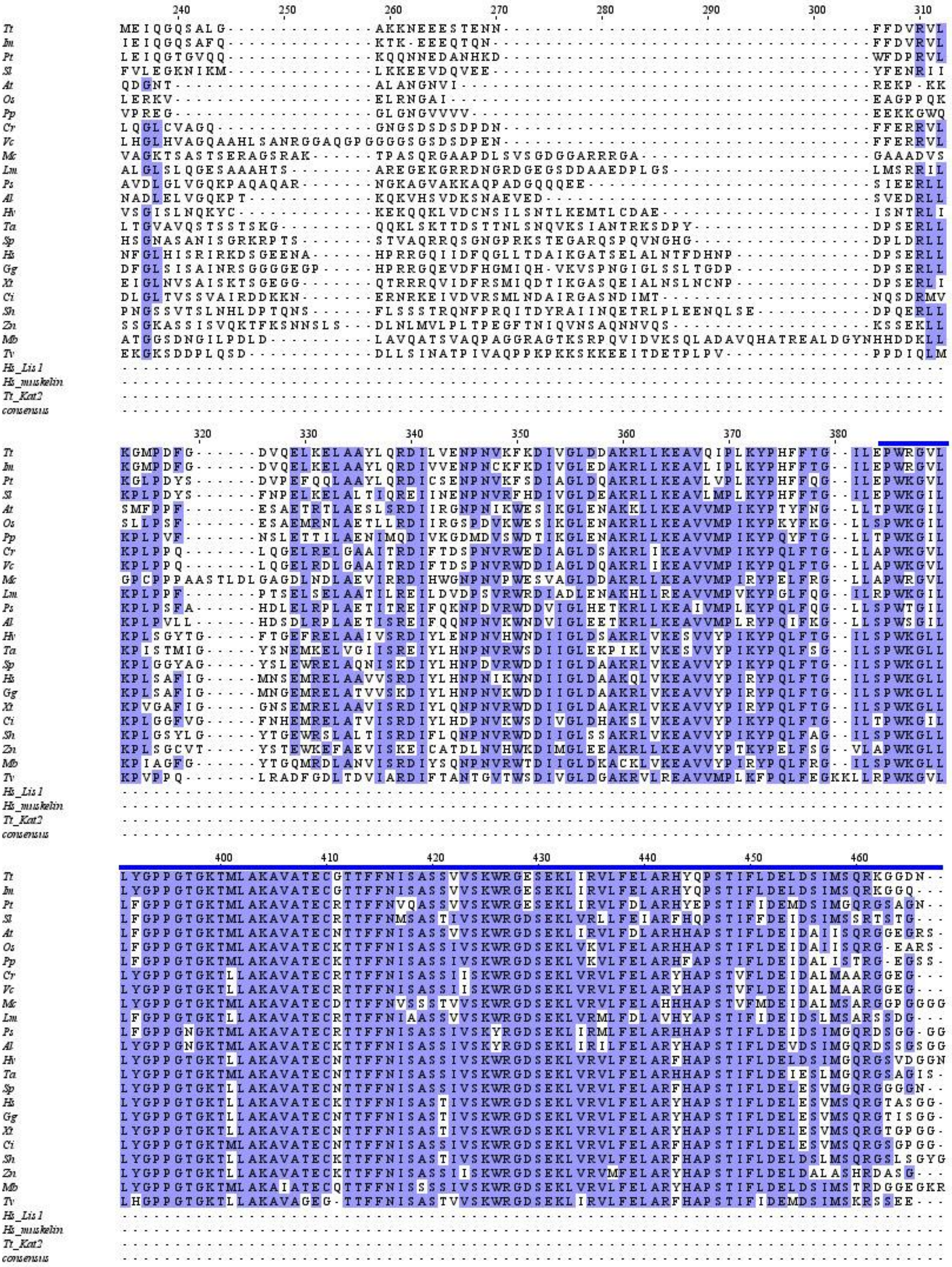
240 250 260 270 280 290 300 310
320 330 340 350 360 370 380
400 410 420 430 440 450 460
Tt
Im
Pt
Sl
At
Os
Pp
Cr
Vc
Mc
Lm
Ps
Al
Hv
Ta
Sp
Hs
Gg
Xt
Ci
Sh
Zn
Mb
Tv
Hs_Lis1
Hs_muskelin
Tt_Kat2
consensus

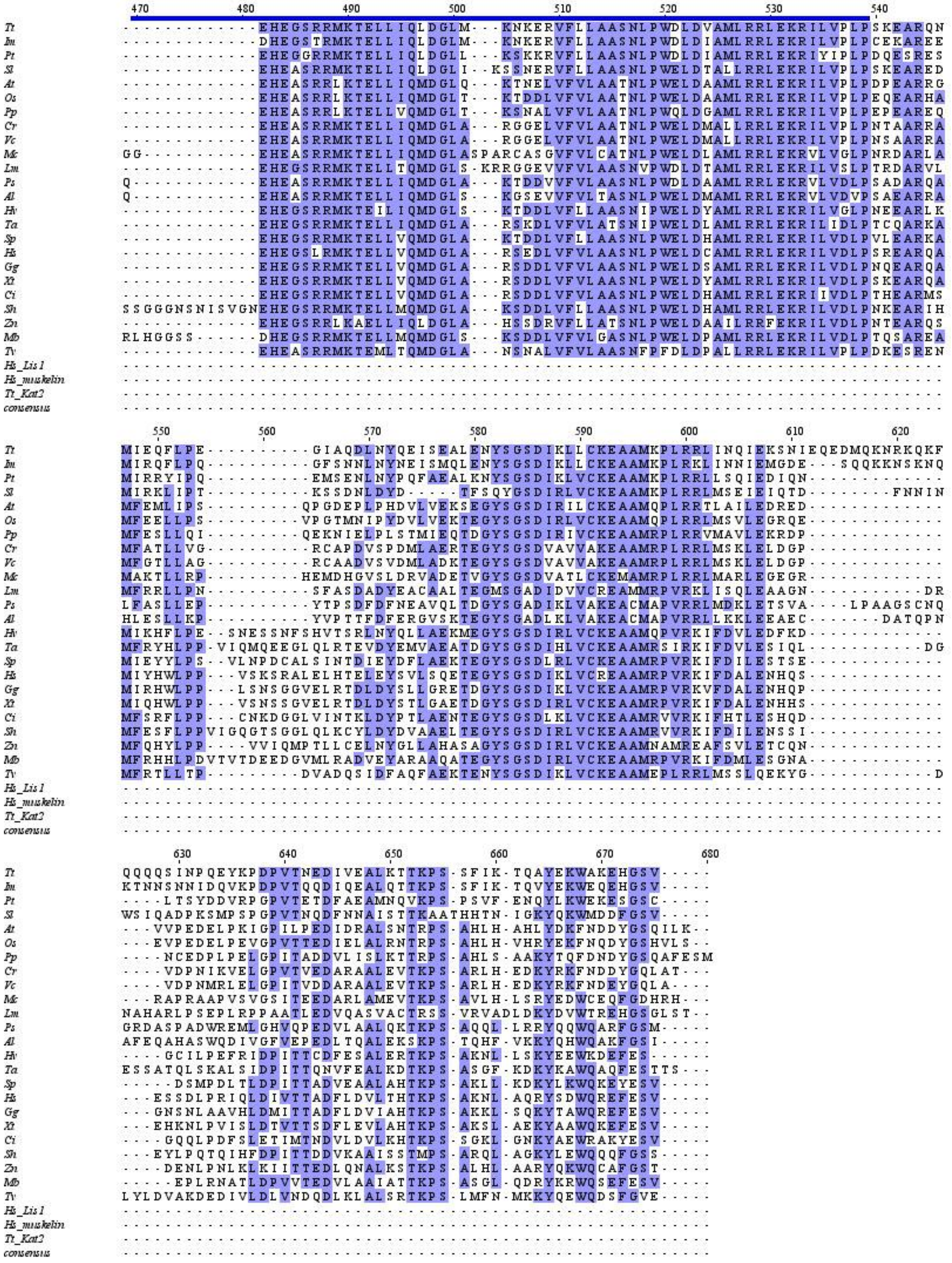

Figure S2. A multiple alignment of Katnal2 ortholog sequences. Al (*Albugo laibachii* Nc14, CCA18838), At (*Arabidopsis thaliana*, NP_973600), Cr (*Chlamydomonas reinhardtii*, XP_001698289.1, corrected), Ci (*Ciona intestinalis*, XP_002130824.1), Gg (*Gallus gallus*, XP_414699.3), Hs (*Homo sapiens*, XP_005258414.1), Hs Miller-Dieker Lissencephaly protein (Lis1) (*Homo sapiens*, AAL34972.1), Hs muskelin (*Homo sapiens*, AAF06698.1), Hv (*Hydra vulgaris*, XP_012554311.1), Im (*Ichthyophthirius multifiliis*, XP_004036585.1), Lm (*Leishmania major strain Friedlin*, XP_001681840.1), Mc (*Micromonas commoda*, XP_002501840.1), Mb

(*Monosiga brevicollis MX1*, XP_001748555.1), Os (*Oryza sativa*, XP_015622246.1), Pt (*Paramecium tetraurelia*, XP_001443881.1), Pp (*Physcomitrella patens*, XP_001761938.1), Ps (*Phytophthora sojae*, XP_009514542.1), Sl (*Stylonychia lemnae*, CDW81059), Sh (*Schistosoma haematobium*, XP_012794975,1, corrected) Sp (*Strongylocentrotus purpuratus*, XP_783887.3), Tt (*Tetrahymena thermophila*, XP_001007193, TTHERM_00414230), Tv (*Trichomonas vaginalis*, XP_001319230.1), Ta (*Trichoplax adhaerens*, XP_002110399.1), Vc (*Volvox carteri f. nagariensis*, XP_002956158.1, corrected), Xt (*Xenopus (Silurana) tropicalis*, NP_001090643.1), Zn (*Zootermopsis nevadensis*, KDR07071.1). An N-terminal fragment and a part of the weakly conserved region of Katnal2 from *Chlamydomonas reinhardtii* (missing in XP_001698289.1 prediction) were reconstructed based on the analysis of the genomic DNA sequence from *C. reinhardtii* v 4.0 database, chromosome 10, contig 53).

The 14 N-terminal amino acids of *Schistosoma haematobium* Katnal2 ortholog were identified about 100 bp upstream of the predicted ATG during genomic DNA analysis using NCBI database. The 95 N-terminal highly conserved amino acids of *Volvox carteri f. nagariensis* were predicted based on the data from blast search of the *Volvox* genomic DNA fragment obtained from NCBI against human proteome.

Note that the alignment also shows the amino acid sequence of a LisH domain identified in human Lis1 (AAA02881.1), human muskelin (AAF06698.1) and *Tetrahymena* Kat2 proteins and a consensus within the LisH domain.

Color lines above the alignment indicate position of: a LisH (red), a CTLH (green) and AAA domain (navy blue) as determined using SMART program. The conserved amino acids were marked using Jalview 2.11.0 program.

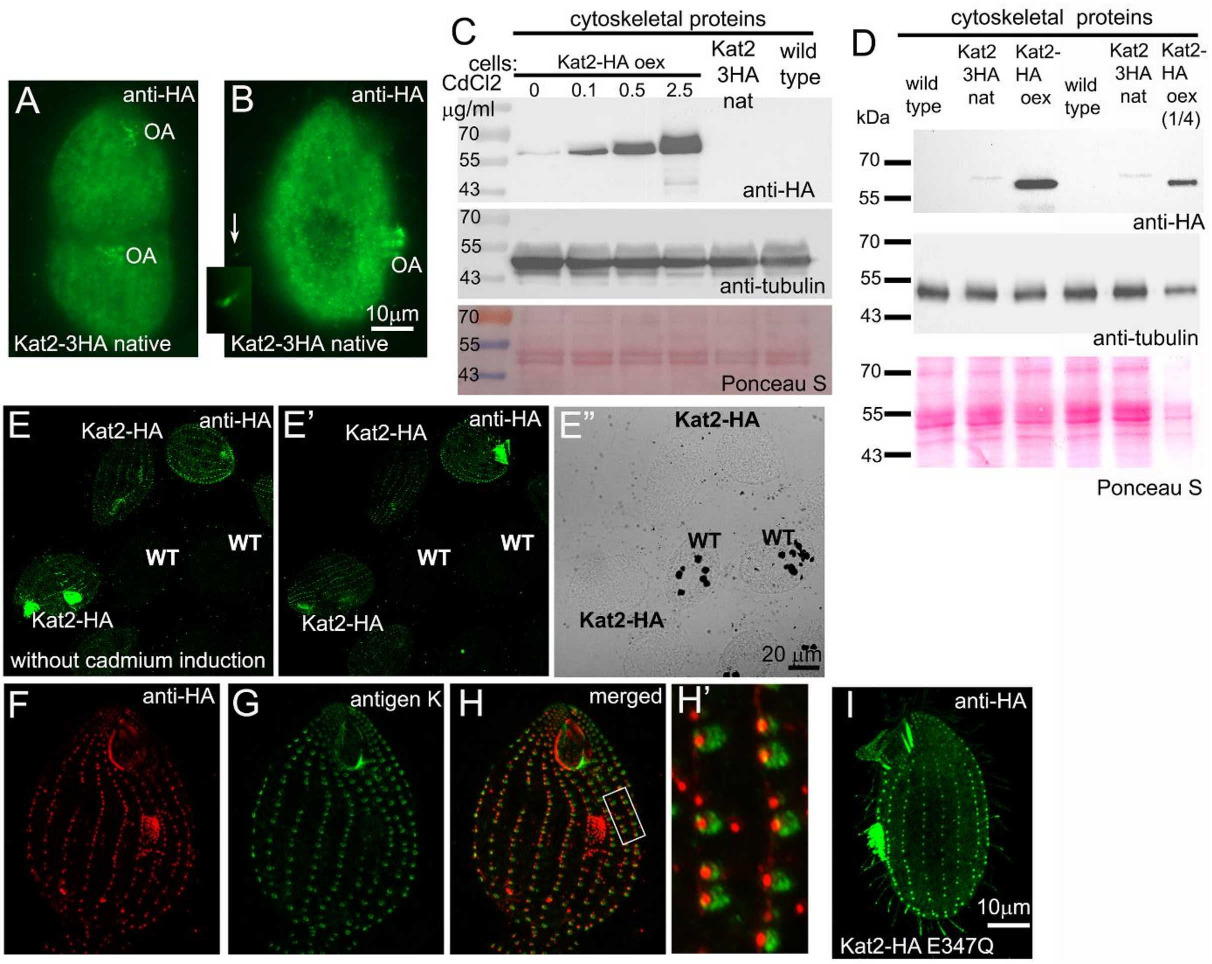

Figure S3. A localization of Kat2-3HA expressed under the control of the native gene promoter. (**a**, **b**) Immunofluorescence confocal images showing presence of Kat2-3HA in short growing cilia of the oral apparatus (**a**) and oral and somatic cilia (**b**), (**c-d**) A Western blot analysis of the levels of Kat2 in the cytoskeletal fraction isolated from cells expressing HA tagged Kat2 either at it native level (nat) or under the control of cadmium dependent *MTT1* promoter (cells were grown with or without (- Cd) cadmium). Tubulin staining (**c**,**d** middle panel) and total protein staining with Ponceau S (**c**,**d** lower panel) are shown as loading controls. (**d**) Note that the native level of Kat2 is significantly lower than in cells harboring *MTT1-KAT2-HA* transgene grown in a medium without cadmium (**d**). Please note that Kat2-3HA expressed under the control of the native promoter migrates more slowly in the gel than the overexpressed Kat2-HA, perhaps due to its posttranslational modification. (**e-e″**) Immunofluorescence confocal images of the mixed population of wild-type cells (WT, cells fed with India ink and thus containing dark food vacuoles) and cells carrying *MTT1-KAT2-HA* transgene grown in a medium without cadmium stained with anti-HA antibodies to compare the intensity of the HA signal. Note that Kat2-HA localizes both, in cilia and near basal bodies. (**f-h′**) Immunofluorescence confocal images of Kat2-HA overexpressing cells stained with anti-HA (**f**) and anti-K antigen (**g**) antibodies. (**h**) Merged image. (**h′**) Magnification of the part of the cell (**h**) marked with a white rectangle. Note that a K antigen is less abundant near new (proximal) basal body of the pair. (**i**) Immunofluorescence confocal image showing a localization of the Kat2-HA (E347Q) with a mutation in the Walker B motif of the AAA catalytic domain. Bar=10 µm.

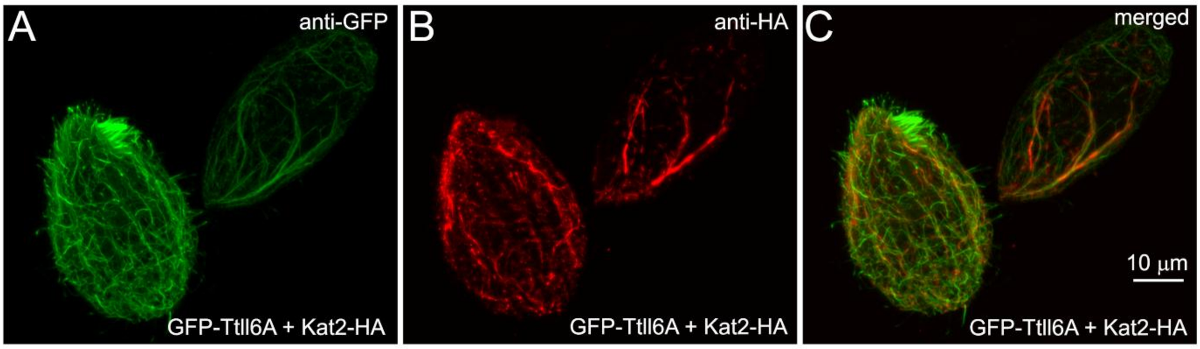

Figure S4. Partial co-localization of the overexpressed GFP-Ttll6A glutamylase and Kat2-HA. *Tetrahymena* cells were grown in SPP medium supplemented with 2.5 μg/ml cadmium chloride for 4 h. Please note that the cell to the right is imaged at the median level. Note also that both GFP-Ttll6A and Kat2-HA highly decorate bundles of subcortical and cytoplasmic microtubules (please compare to Janke et al., 2005, Wloga et al., 2010 [23,24]). (**a**) GFP-Ttll6A, (**b**) Kat2-HA, (**c**) merged image.

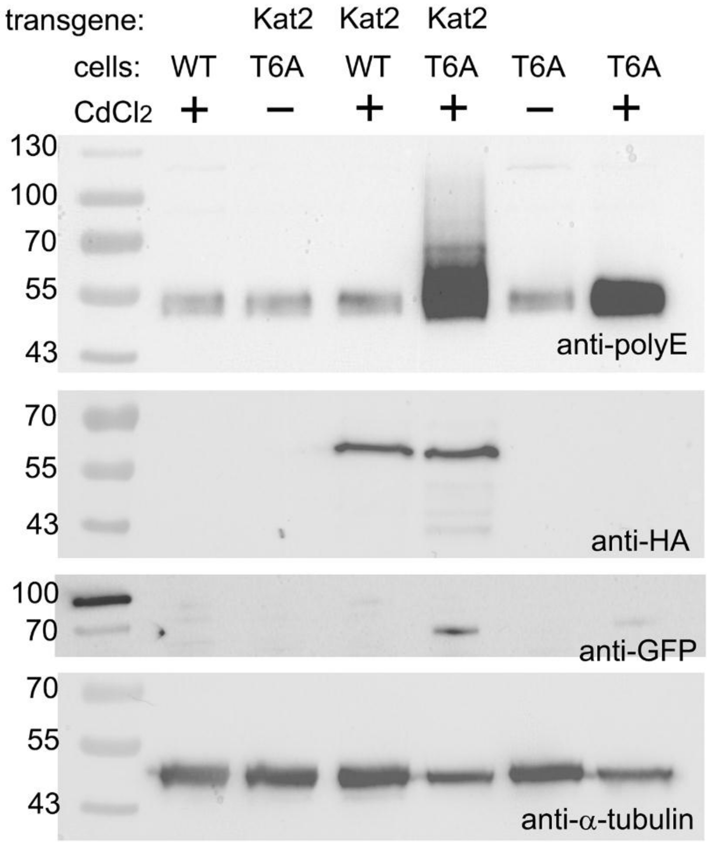

Figure S5. Comparative Western blot analysis of the level of tubulin glutamylation in cells co-overexpressing Kat2-HA and tubulin glutamylase, GFP-Ttll6A. Cytoskeletal fraction was isolated from wild-type cells, cells carrying transgenes enabling expression of either Kat2-HA or GFP-Ttll6A glutamylase, and cells co-overexpressing both proteins. Cells were either non-induced ($CdCl_2$ "-") or induced ("+") with 2.5 μg/ml cadmium chloride for 4 h. To detect Kat2-HA and GFP-Ttll6A, 20 μg of cytoskeletal proteins was loaded. To detect α-tubulin and glutamylated tubulin, 5 μg of cytoskeletal proteins was loaded.

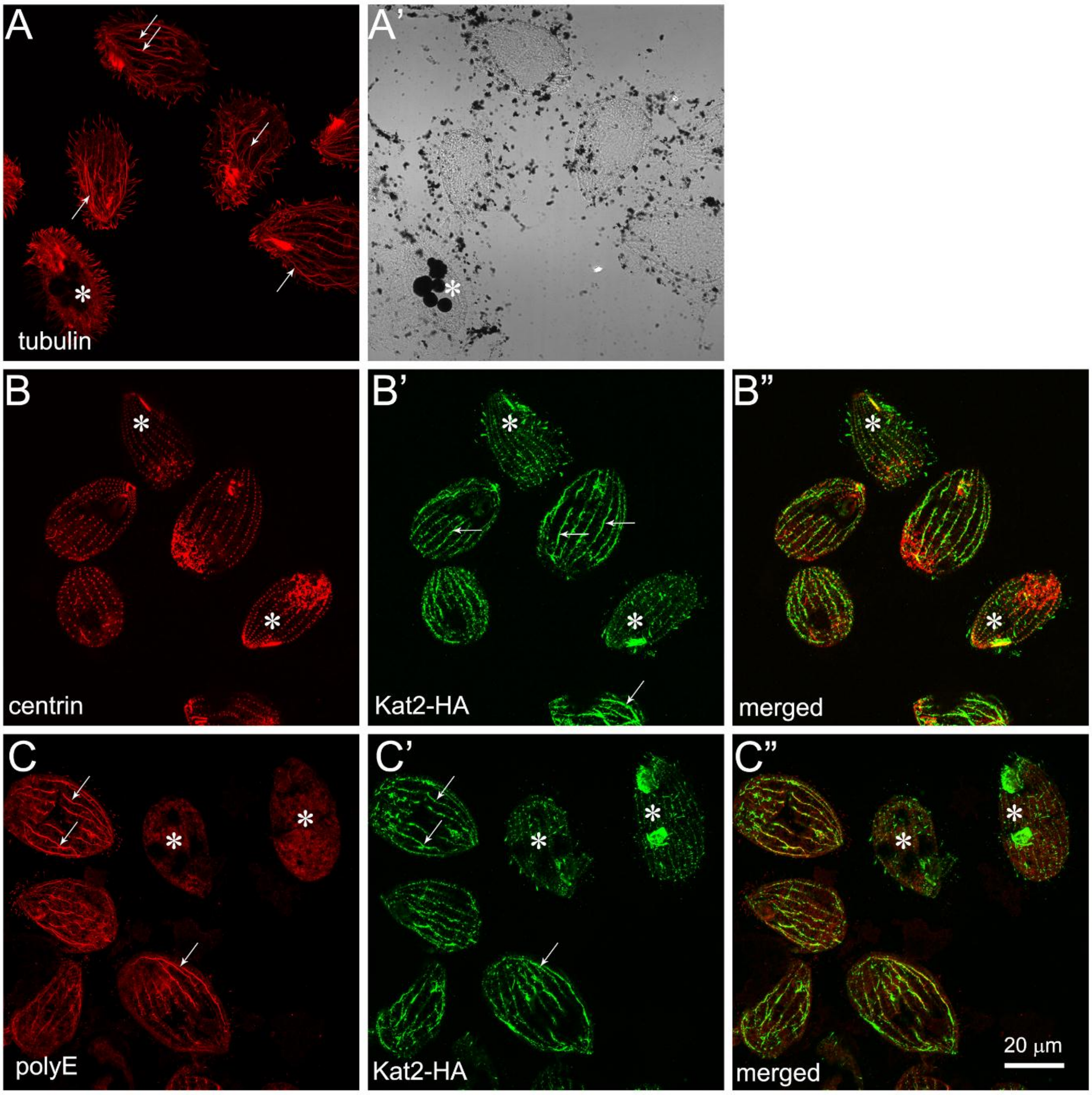

Figure S6. The elevated level of tubulin glutamylation in cells co-overexpressing Kat2-HA and GFP-Ttll6A alters Kat2-HA localization. Immunofluorescence confocal images of the mixed population of cells overexpressing Kat2-HA alone (indicated by the presence of dark food vacuoles and by white star) or co-overexpressing Kat2-HA and GFP-Ttll6A and thus having highly glutamylated microtubules. (**a-a′**) Cells stained with 12G10, anti-α-tubulin antibodies to visualize cilia; note a presence of the bundles of cortical and subcortical microtubules (some are indicated by white arrows) in cells overexpressing GFP-Ttll6A glutamylase. (**a′**) A corresponding phase contrast image showing which cell has food vacuoles (left lower corner). (**b-c″**) Immunofluorescence confocal images of cells stained with either anti-centrin antibodies (**b**) to visualize basal bodies or polyE antibodies (**c**) to show presence of the glutamylated microtubules and co-stained with anti-HA antibodies (**b′, c′**) to localize Kat2-HA. (**b″, c″**) Merged images. The centrin localization (**b**) is not altered in cells with normal and elevated levels of tubulin glutamylation. Please note a presence of bundles of highly glutamylated cortical and subcortical microtubules (some are indicated by white arrows) in cells overexpressing GFP-Ttll6A glutamylase (**c**) and re-localization of Kat2-HA in these cells (**c′**). Note also that in GFP-Ttll6A overexpressing cells Kat2-HA is no longer visible in cilia but instead co-localizes with bundles of microtubules (some are indicated by white arrows). Bar=20 μm.

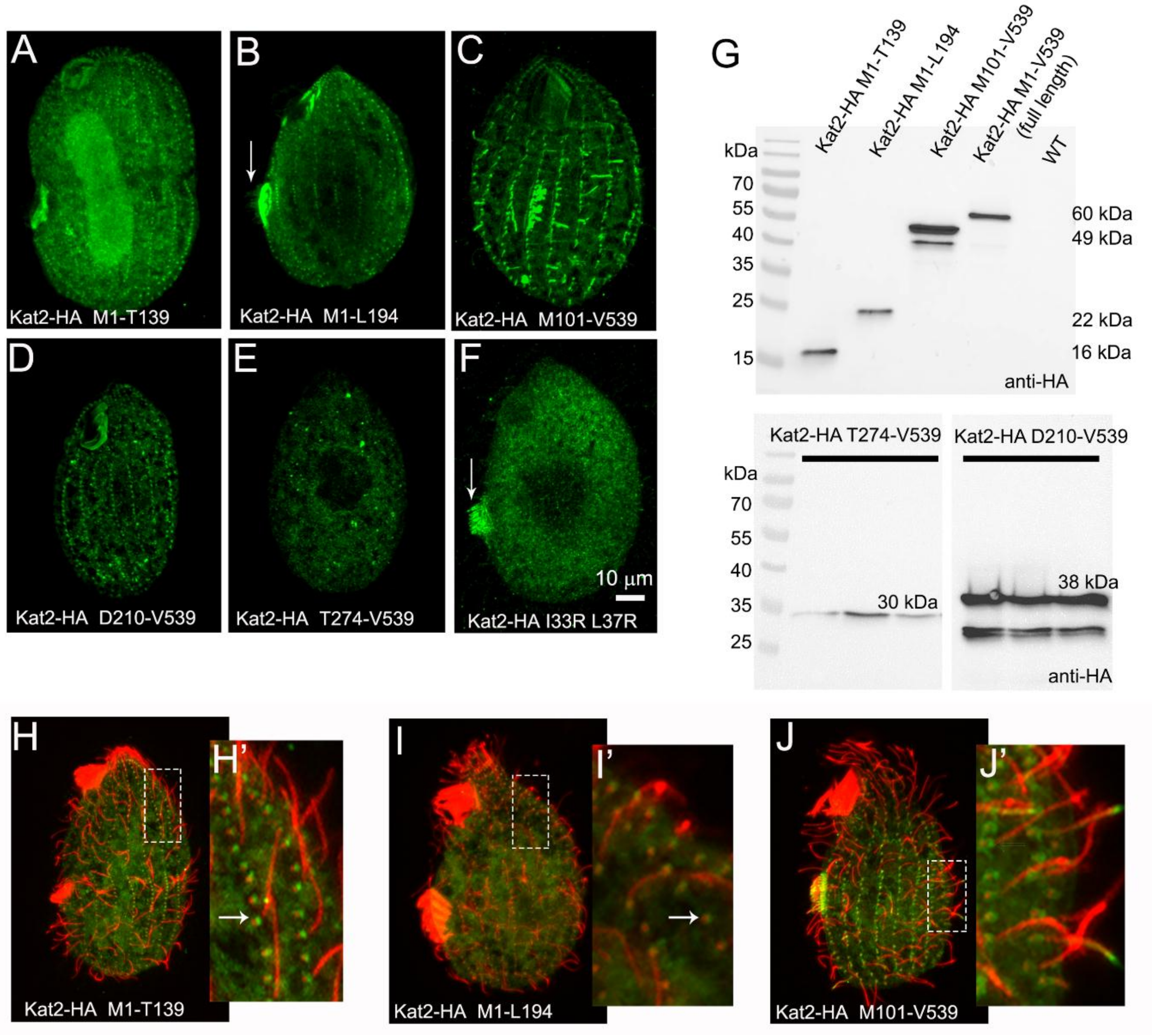

Figure S7. Immunolocalization of Kat2-HA truncations. (**a-f**) Immunofluorescence confocal images of *Tetrahymena* cells carrying a transgene enabling overexpression of the C-terminally HA-tagged truncated variants of Kat2: (**a**) M1-T139, (**b**) M1-L194, (**c**) M101-V539, (**d**) D210-V539 or (**e**) T274-V539. (**f**) Cell overexpressing Kat2-HA with double mutation within a LisH motif (I33R, L37R); note a presence of the protein in growing cilia (white arrow); a presence of the mutated Kat2 near the basal bodies is obscured by the overexpressed protein accumulated in the cytoplasm. Before fixation, cells were induced for 3 h with 2.5 μg/ml $CdCl_2$. Note that the truncations containing LisH and CTLH localize near basal bodies (**a, b**), while a lack of these fragments causes partial Kat2 redistribution (**c**). Bar =10 μm. (**g**) A Western blot analysis of the cytoskeletal fraction isolated from cells overexpressing Kat2 truncations. Numbers to the right (upper panel) or above the bands (lower panel) indicate the calculated molecular mass of the Kat2 truncation. (**h-j′**) Immunofluorescence confocal images of *Tetrahymena* cells overexpressing (**h-h′**) Kat2-HA M1-T139, (**i-i′**) Kat2-HA M1-L194, (**j-j′**) Kat2-HA M101-V539, double labeled with anti-HA and GT335 antibodies recognizing glutamylated tubulin in cilia and basal bodies. (**h′, i′, j′**) Magnification of the part of the cell marked with a white rectangle (**h, i, j**).

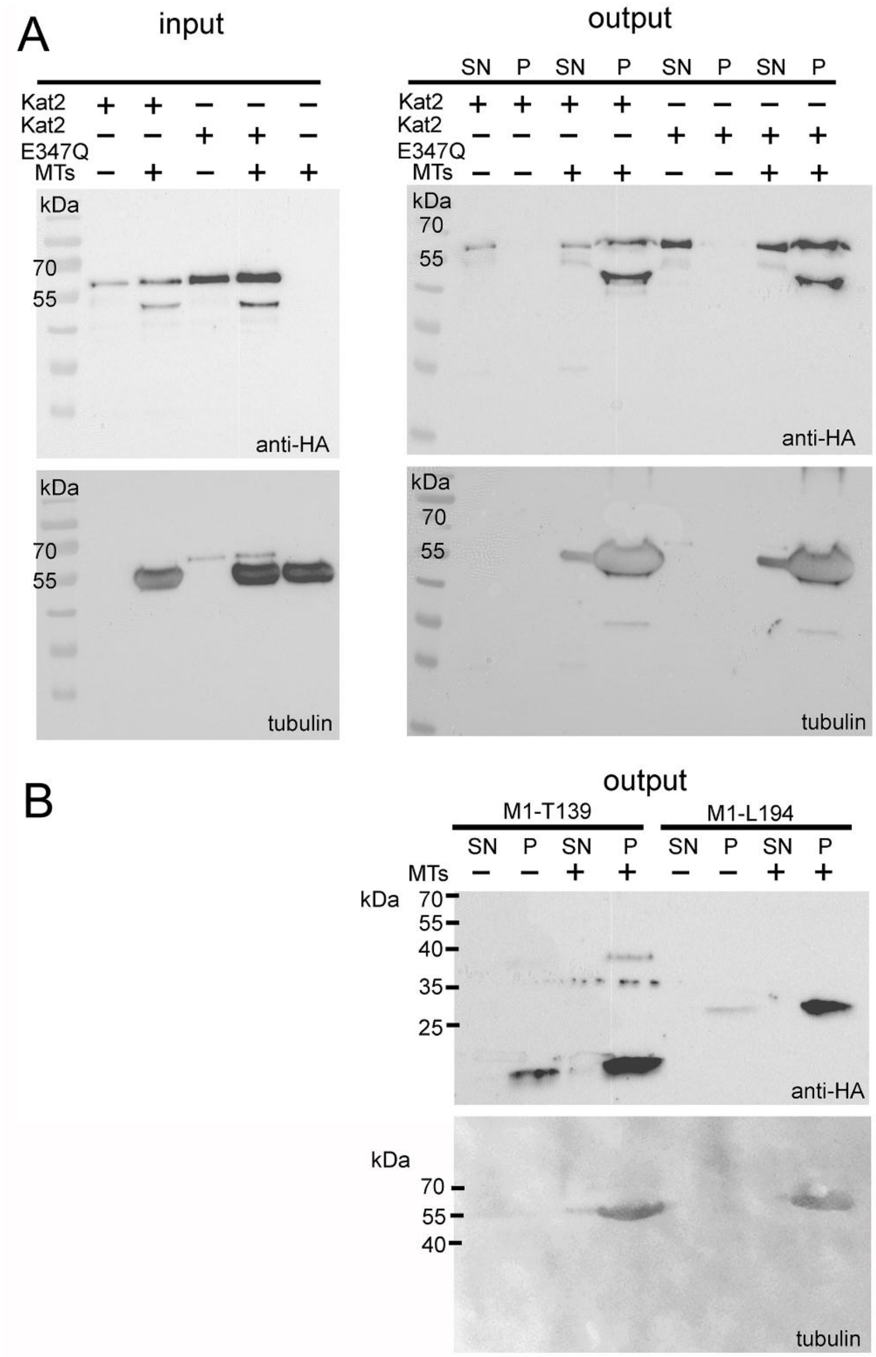

Figure S8. An in vitro microtubule binding assay. (**a, b**) A Western blot analysis of the Kat2-HA or Kat2-HA E347Q isolated from the cytosolic fraction of the overexpressing cells and incubated with in vitro polymerized microtubules. The panel to the left shows proteins just after mixing (input) while the panel to the right shows the same samples after 30 min. of incubation. Note that after incubation with microtubules both Kat2-HA and Kat2-HA E347Q were in the pellet fraction suggesting binding to microtubules (**a**) while some Kat2-HA M1-T139 and Kat2-HA M1-L194 pelleted even without microtubules (**b**).

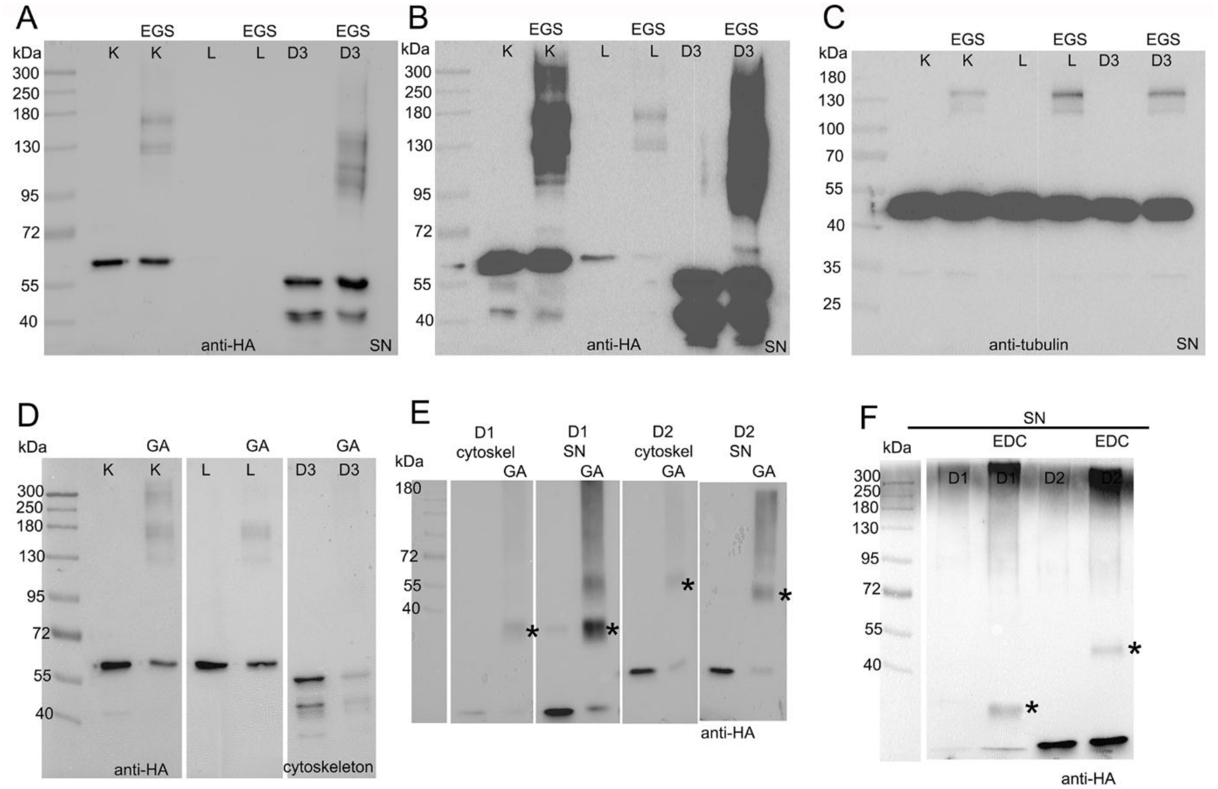

Figure S9. LisH domain plays a role in the formation of Kat2 complexes. (**a-c**) A Western blot-based identification of the HA-positive (**a**, **b**) and tubulin-positive (**c**) in vivo-formed and EGS-stabilized complexes in supernatant (SN) isolated from *Tetrahymena* cells overexpressing for 4 h Kat2-HA (K), Kat2-HA I33R L37R (L), or Kat2-HA M101-V539 (D3). (**b**) The same as (**a**) but with longer exposure to detected Kat2-HA I33R L37R. (**a, b**) Note that complexes formed by Kat2-HA M101-V539 are smaller compared to those formed in cells overexpressing a full-length protein while tubulin-positive complexes are of similar size in all analyzed samples. Note also that Kat2-HA I33R L37R may form complexes although the level of the mutated protein is significantly lower in the cell. (**d-f**) The Western blot-based identification of the HA-positive complexes stabilized in vitro by 0.02% glutaraldehyde (**d, e**) or EDC (**f**). Stars in (**e**) and (**f**) indicate bands of the size corresponding to the molecular mass of the dimer formed by Kat2 truncation; Kat2 M1-T139 (D1) and Kat2 M1- L194 (D2).

Table S1

Primers used to amplify fragments of the genomic DNA. The nucleotide sequence of the introduced restriction site is underlined. The ATG or TGA are in bold. In red are marked changes introduced in the nucleotide sequence.

Amplified fragment	Forward primer	Reverse primer
KAT2 ORF	AATT ACGCGT T **ATG**AGTTATCTACTATCAAAAGTCCGC	AATT GGATCC AACTGAACCATGTTCCTTAGCC
KAT2 M1-T139	AATT ACGCGT T **ATG**AGTTATCTACTATCAAAAGTCCGC	AATT GGATCC TGTTGTAT TTAAAT TGCTGT TCACG
KAT2 M1-L194	AATT ACGCGT T **ATG**AGTTATCTACTATCAAAAGTCCGC	AATT GGATCC TACAAGAGCTGATTAACCCTAAATTTC
KAT2 M101-V539	AATT ACGCGT T **ATG** CCATCTTTTCCGAAGATATC	AATT GGATCC AACTGAACCATGTTCCTTAGCC
KAT2 D210-V539	AATT ACGCGT T **ATG** GAT GTT AGA GTA TTA A AAGGAA TGC	AATT GGATCC AACTGAACCATGTTCCTTAGCC
KAT2 T274-V539	AATT ACGCGT T ATG ACT GGA ATA TTA GAA C CTTGG	AATT GGATCC AACTGAACCATGTTCCTTAGCC
LisH domain mutagenesis	GCTTCGTTTGAGATATCGTACAAACATTGG G TATATAGAATCATCTT	TGTACGATATCTCAAACGAAGCACTAT TAAGTTTCTTTTACGATCA
E347Q mutagenesis	ATTTTCTTAGATCAGCTGGATTCTATTATGT CG	CGACATAATAGAATCCAGCTGATCTAAGAAAA T
Native locus ORF	AATT ACGCGT T **ATG**AGTTATCTACTATCAAAAGTCCGC	AATT GGATCC AACTGAACCATGTTCCTTAGCC
Native locus 3'UTR	AATT CTGCAG CTATTTTAAGATACCTTAGAAAAGCAC	AATT CTCGAG GATCCAGTATAAAACTCAAACCG
GRL4 fragment	AATT CCGCGG TT GAA GCT GAT TAA GGC AAG AA	AATT AGATCT ACTTAGGAGCACTCAAAACTTCA
GRL3 fragment	AATT GATATC TGC TGG ATA AAG TGC TGG TAG	AATT CTCGAG GTTAATGTTTTCAACAGCAGGAAC

Table S2

Proteins that co-immunoprecipitate with Kat2-HA. X/Y – number of total and specific peptides identified. In experiment 1 and 2 total immunoprecipitate was analyzed. In experiment 3, the immunoprecipitate was run on the 8% SDS-PAGE gel and silver stained bands were excised and analyzed.

protein	TGD number	Exp1	Exp 2	Exp2-Ctr	Exp 3
Kat2	TTHERM_00414230	54/26	35/20	0	204/55
α-tubulin ATU1	TTHERM_00558620	6/4	1/1	0	23/16
β-tubulin BTU1	TTHERM_00348510	15/13	9/9	0	26/19

TGD – Tetrahymena Genome Database
